# Demographic subgroup-aware analysis of blood pressure predictive models

**DOI:** 10.1371/journal.pdig.0001687

**Published:** 2026-10-15

**Authors:** Blessing Kolawole, Sinuo Fan, Yingjie Lao, Valencia J. Koomson

**Affiliations:** 1 Department of Computer Science, Tufts University, Medford, Massachusetts, United States of America; 2 Department of Electrical and Computer Engineering, Tufts University, Medford, Massachusetts, United States of America; Chinese PLA General Hospital, CHINA

## Abstract

Blood pressure (BP) is an important cardiovascular health indicator, usually monitored non-invasively with a sphygmomanometer, which can be bulky and inconvenient for real-time monitoring. Blood pressure estimation using photoplethysmography (PPG) waveform analysis offers a promising alternative for intermittent or continuous cuff-less BP monitoring. However, changes in vascularization due to individual physiology and age can directly affect the morphology of the PPG waveform. Thus, prediction accuracy requires a demographic-aware approach. To address these challenges, we trained three conventional ML models for BP prediction and conducted a subgroup-aware analysis to evaluate model performance across individual and intersectional demographic groups. Additionally, we show that signal quality assessment is a pivotal step in the signal-processing pipeline before feature extraction to mitigate external perturbations (e.g., motion artifacts) in the PPG waveform. We achieve a mean absolute error (MAE) of **3.36 mmHg** for systolic blood pressure (SBP) and **1.76 mmHg** for diastolic BP (DBP) with models trained when signal quality assessment is incorporated.

## Author summary

Recent advancements in machine learning and artificial intelligence have led to the development of numerous predictive models to derive blood pressure estimates from PPG signals. Generally, the performance of blood pressure (BP) prediction models is reported as an average across the entire dataset without accounting for the distribution of different populations within the data. However, recent studies have highlighted significant disparities in the performance of machine learning models across diverse populations, raising concerns about equity and fairness. While many of these studies are not directly related to blood pressure prediction models, conducting such evaluations in this domain is crucial to ensure the robustness and equity of novel BP estimation methods that could be widely adopted. Dataset bias can arise from various distinct biases embedded in data-generating processes. Certain biases, including representation bias along demographic lines, can result in skewed outcomes. Performance disparities can stem from various factors, including differences in demographic characteristics, data quality, and model training methodologies. This paper aims to examine disparities in blood pressure prediction models, identifying key factors contributing to inconsistencies and enhancing model performance across diverse populations.

## 1. Introduction

Hypertension is a critical medical condition impacting the global population, and it stands as a significant contributor to morbidity and mortality rates [1]. Traditional approaches to monitoring hypertension involve measuring blood pressure using invasive techniques such as arterial catheterization and cuff-based non-invasive methods like auscultation and oscillometry [2,3]. Cost and inconvenience for sustained real-time monitoring are significant drawbacks to these approaches within non-clinical settings, substantiating the demand for cuffless non-invasive methodologies. Given the similarity between arterial blood pressure and photoplethysmogram waveforms, blood pressure (BP) estimation using photoplethysmography (PPG) signals has gained significant attention in recent years [4–8].

Various studies have explored different machine-learning techniques to enhance prediction accuracy. One such study by [9] conducted a comprehensive analysis to determine the optimal feature extraction techniques and machine learning algorithms for BP estimation. Their findings indicate that time-domain-based features yield better accuracy than other extraction techniques. [10] constructed time series from morphological PPG features and pulse transit time values, dividing them into high- and low-frequency components before feeding them into a combination of DNN and RNN architectures for BP estimation. [11] proposed a parallel architecture consisting of three residual neural networks (ResNet) to predict BP using the PPG waveform and its first and second derivatives. [12] developed a Siamese convolutional neural network (CNN) using PPG spectrograms to predict BP variations relative to a calibration value. Furthermore, for the correct estimation of the BP values, signal quality evaluation is critical; therefore, various approaches have been suggested to evaluate the quality of PPG signals [13–15], often starting with checks for physiological plausibility to pinpoint segments of poor quality, followed by more sophisticated methods to identify subtle distortions [16]. [17,18] assessed PPG signal quality based on key characteristics derived from pulse waves, such as pulse wave amplitude, perfusion index, and timing metrics like systolic phase duration and inter-beat intervals, which are classified as high or low quality using predefined thresholds or machine learning models. [19] used shape-related features, including diastolic peaks, zero-crossing rate, and signal-to-noise ratio, and higher-order statistics like skewness, kurtosis, and Shannon entropy to identify noise-related outliers and quantify signal disorder. Other methodologies, including pulse wave similarity analysis and autocorrelation, have been introduced to enhance assessment accuracy [16,20]. They use template-matching techniques, which involve comparing individual pulse waves to an average template using metrics like Euclidean distance or correlation coefficients [21].

[22] reviewed eleven state-of-the-art machine learning (ML) models and classified them into three main categories: (1) signal-to-signal (Sig2Sig) models, which generate continuous arterial blood pressure (ABP) signals from continuous PPG signals [7,23], (2) signal-to-label (Sig2Lab) models, which directly map PPG signals to BP values, and (3) feature-to-label (Feat2Lab) models, which utilize handcrafted PPG features for BP estimation. Our study aligns with the Feat2Lab approach, focusing on extracting the optimal set of features for prediction of SBP and DBP values. While most studies show improved performance [5,6,24,25], they predominantly train models and report performance results on an overall population without considering patient sub-populations or demographic differences. [26–28] highlight the risks associated with such approaches, as they may overlook critical disparities in disease manifestation among different demographic groups. To address these challenges, we trained three conventional ML models for BP prediction and conducted a subgroup-aware analysis to evaluate model performance across individual and intersectional demographic groups. Additionally, we show that signal quality assessment is a pivotal step in the signal processing pipeline (Fig 1) before feature extraction to mitigate against external perturbations (e.g., motion artifacts) in the PPG waveform.

**Fig 1:**
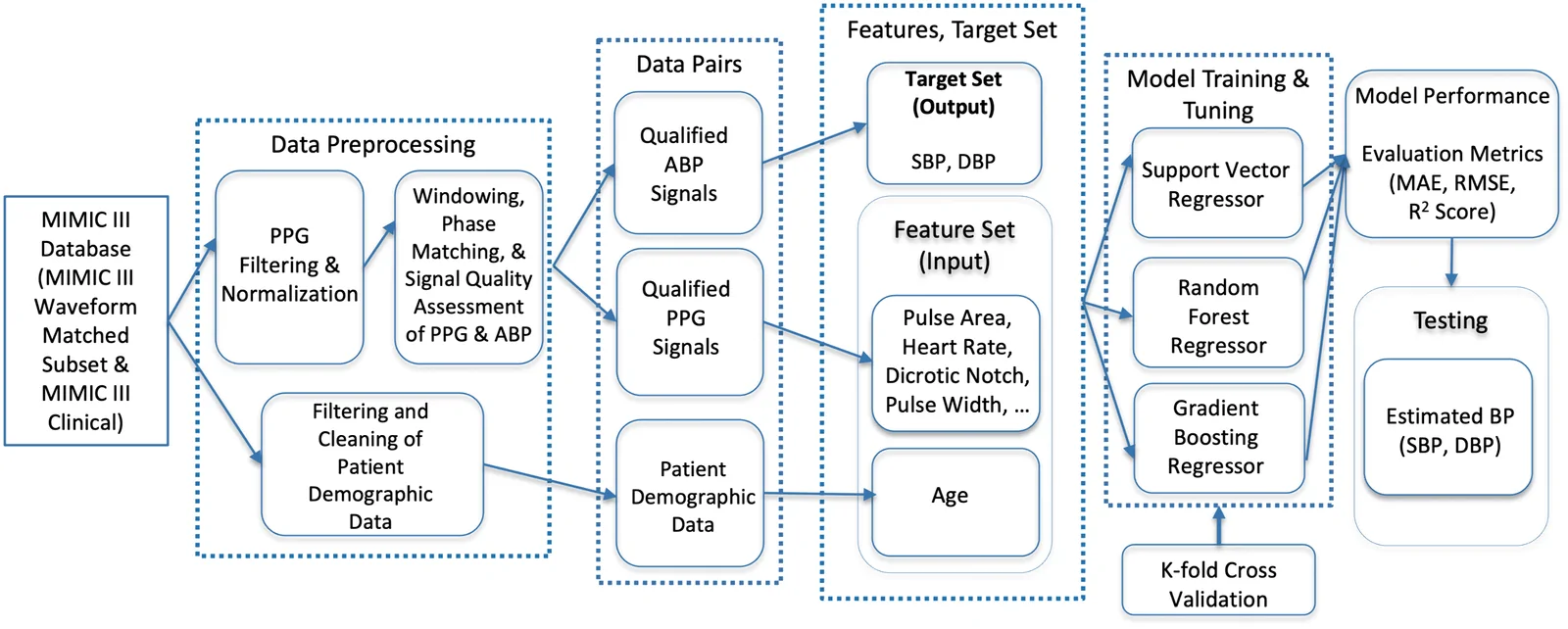
Data preprocessing and machine learning model training pipeline.

## 2. Materials

### 2.1 Institutional Review Board (IRB)

The data used in this research work are publicly available and do not require IRB approval.

### 2.2 Data

We obtained physiological waveform data such as arterial blood pressure (ABP) and photoplethysmogram (PPG, commonly labeled ’PLETH’), from the publicly available MIMIC-III Waveform Matched Subset Database (M3MSWDB), which contains high-resolution recordings of multiple physiologic signals collected from bedside patient monitors in intensive care units (ICUs). For each subject, the waveform recordings are linked to the corresponding demographic information extracted from the clinical records available in the MIMIC-III Clinical Database (M3CDB). This integrated dataset provides comprehensive information for analyzing correlations between subjects’ physiological measurements, demographic information (age), and blood pressure estimates. It also allows performance analysis by demographic factors.

### 2.3 Cohort selection

This study cohort includes selected adult patients aged between 18 and 89 years. We included only patients with both arterial blood pressure (ABP) and photoplethysmogram (PPG) signals, and recordings had to be at least 10 seconds long (a minimum of 1250 samples at a typical sampling rate of 125 Hz). Additionally, the waveform recordings had to be accurately matched to the MIMIC-III clinical database, requiring valid entries for both subject_id and icu_intime. After applying this restriction, Fig 2 shows our final cohort of 2315 patients; we describe its summary statistics in Table 1.

**Fig 2:**
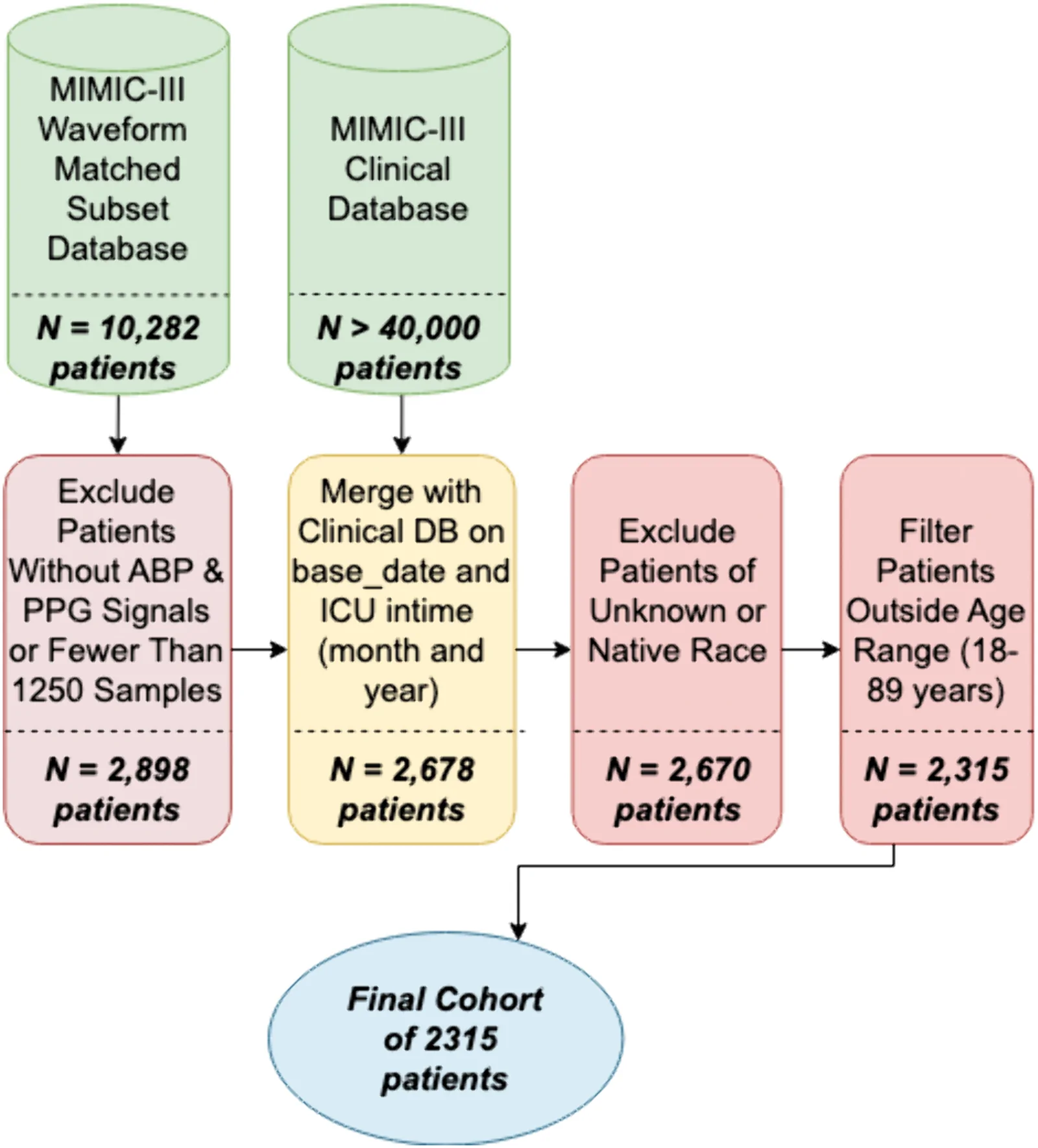
Cohort selection from M3MSWDB and M3CDB.

**Table 1:** Selected cohort summary by demographic subgroups, including race, age, and gender. Granular details on racial categorization are described in Supplementary material [Table J in S1 Appendix]. Disease history is reported as the number and percentage of patients with such a condition.

Cohort Summary Statistics			
Variables	Category	No. of Subjects	Percentages
Gender	Male	1327	57.32%
	Female	988	42.68%
Age Groups	18–25	40	1.73%
	26–35	90	3.89%
	36–45	164	7.08%
	46–55	374	16.16%
	56–65	502	21.68%
	66–89	1138	49.16%
Ethnicity	Asian	75	3.24%
	Black	207	8.94%
	Hispanic	103	4.45%
	White	1813	78.32%
	Other	115	4.97%
Disease history	Acute kidney failure	522	22.5%
	Acute respiratory failure	595	25.7%
	Atrial fibrillation	679	29.3%
	Congestive heart failure	632	27.3%
	Coronary atherosclerosis of native vessel	491	21.2%
	Hyperlipidemia	629	27.2%
	Hypertension	1078	**46.6%**
	Severe sepsis	391	**16.9%**
	Type II diabetes mellitus without complication	486	21.0%

## 3. Methods

As mentioned previously, this study uses data from the MIMIC III waveform matched subset database 2.2. The dataset was obtained from patients in the intensive care unit (ICU) and may not be representative of real-world population demographics. With this in mind, we structure this section to discuss (1) the demographic characteristics of the dataset, (2) our blood pressure prediction model set-up, and (3) the assessment of performance across demographic subgroups, including race, gender, age, and their intersections (e.g., race-age combinations). Additionally, we discuss the impact of including signal quality assessment in the signal processing pipeline on model performance. In one approach, we included only signals meeting a signal quality index (SQI) threshold of 70% for feature extraction; in the other, we omitted signal quality assessment and used all available signals.

### 3.1 Demographic distribution and biases

The dataset used in this study is inherently biased in terms of racial and age distribution. Approximately 78% of the dataset consists of individuals who identify as White. The remaining 22% is distributed among Asian, Black, Hispanic, and other racial/ethnic groups as shown in Table 1. This imbalance may influence the generalizability of the results, particularly when applying models trained on this dataset to more diverse populations [29].

Age-wise, the dataset is skewed toward an older population, as shown in Fig 3 and Table 1. 70% of the data is derived from individuals aged 56–89, while the remaining 30% comprises participants aged 18–55. This demographic distribution is an important factor in analyzing trends related to blood pressure, as age significantly influences cardiovascular health and blood pressure variability.

**Fig 3:**
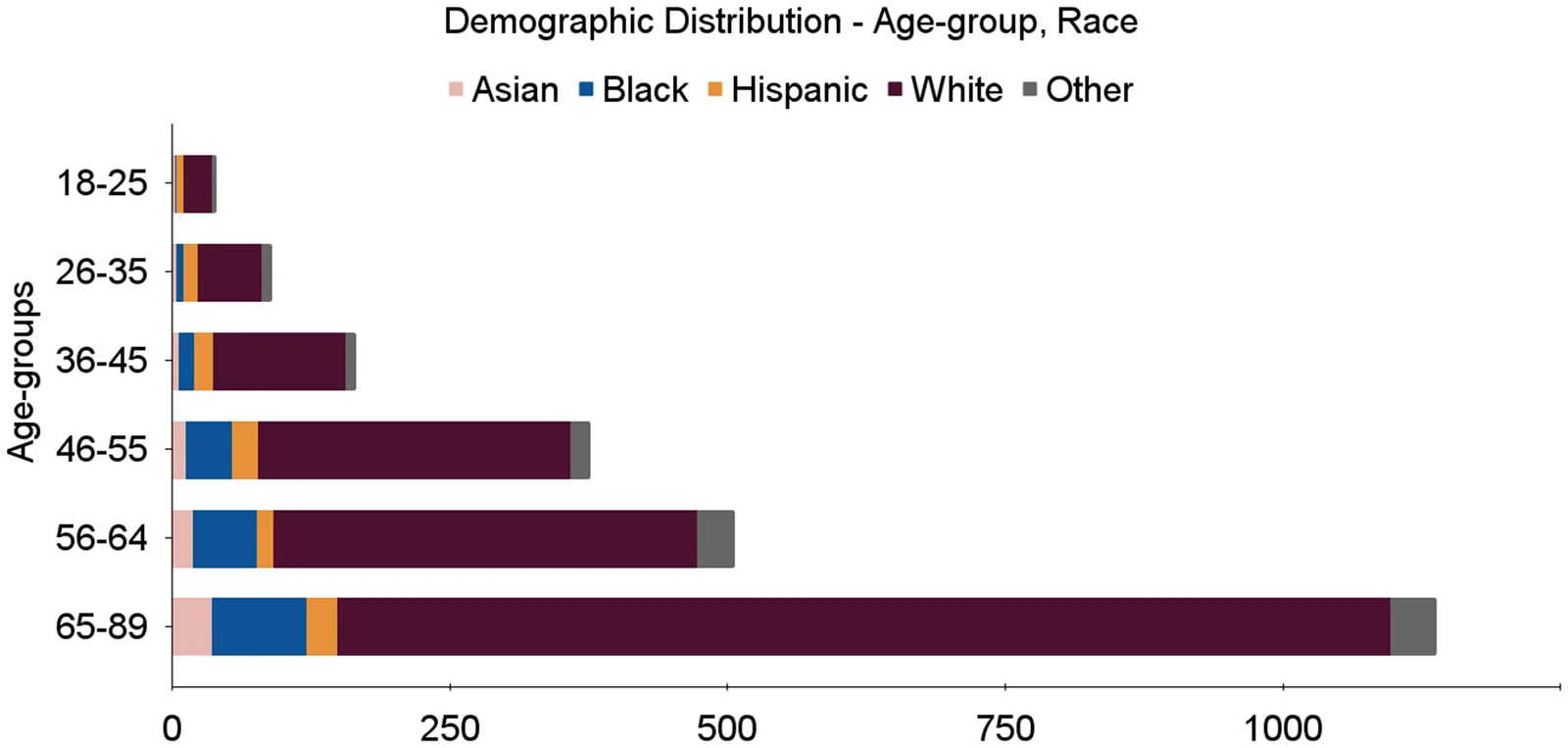
Selected cohort demographic distribution by age-group and race. The dataset displays a significant demographic imbalance, with 70% of individuals aged 56–89 and about 78% White. Because it is sourced from ICU patients, this skew may reflect disparities in healthcare access rather than the general population.

### 3.2 Signal processing

Signal processing techniques applied to prepare the raw PPG and ABP waveform data include digital filtering, which uses low-pass and/or high-pass filters to remove noise in the signal; window phase matching of PPG and ABP signals using cross-correlation to correct for physiological delays due to differing extraction points, limiting the shift to a maximum of ±1 second to prevent unrealistic adjustments. After alignment, both signals were segmented into 10-second windows with a 50% overlap; then, we normalized the records using z-score normalization. Finally, all records are parsed in the signal quality phase to ensure data quality. We describe these processing steps in detail below:

1. Digital Filtering: The PPG signal is filtered to eliminate irrelevant frequency content [30], such as high-frequency noise or low-frequency baseline wander [31]. We eliminated low-frequency noise in the PPG signal using a 4th-order Butterworth filter with a frequency of 5Hz. This low-pass filtering is a compromise between retaining the original signal features and increasing robustness to noise [32].
2. Window Phase Matching: This refers to a temporal alignment procedure in which fixed-length windows of PPG and ABP signals are synchronized in time before feature extraction. For each analysis window, the PPG waveform is phase-aligned with the corresponding ABP segment, ensuring that extracted PPG morphological features (e.g., pulse width, systolic and diastolic waveform descriptors) are correctly mapped to their true systolic and diastolic pressure labels derived from ABP.
3. Segmentation: Each patient had multiple recorded sessions of PPG and ABP signals. The raw PPG signal for each patient was segmented into multiple 10-second windows using a sliding-window approach with 50% overlap; each resulting non-zero segment is referred to as a *record*. During this process, the PPG signal was phase-matched with the corresponding ABP signal as described in (2) above, and the relevant ABP segment for each window was extracted. A segment is excluded from the dataset if the signal quality index (SQI) of the PPG or ABP signal in any window is less than the threshold described in Section 4.

For each valid PPG segment, the reference SBP and DBP values were derived directly from the ABP waveform within the same segment using a peak detection algorithm. SBP was computed as the median of the maximum pressure points (peaks) in the ABP waveform corresponding to each cardiac cycle, representing the peak arterial pressure during systole. DBP was identified as the median of the minimum pressure points in the ABP waveform, occurring just before the onset of the next systolic phase, representing the lowest arterial pressure during diastole. Multiple plausibility checks ensured data quality: BP values were restricted to physiologically valid ranges (SBP: 75–165 mmHg, DBP: 40–120 mmHg), and median heart rates outside 50–140 bpm were discarded.

The statistical distribution of SBP and DBP values across the dataset is illustrated in Fig 5.

4. Normalization: Z-score normalization standardizes the PPG signal by directly adjusting its mean and variance. Each data point is transformed by subtracting the mean and dividing by the standard deviation of the dataset using the formula: $z=\frac{x-\mu }{\sigma }.$ In this equation, *x* represents the raw signal value, μ is the mean, and σ is the standard deviation of the signal. This transformation centers the data at a mean of 0 and scales it to a standard deviation of 1, ensuring uniformity across all records.
5. Signal Quality Assessment: We perform a beat-to-beat cross-correlation of all records to analyze their quality [18]. First, we divide each record into individual beats by identifying local minima; we then align each successive beat at its peak for comparison, as shown in Fig 4. We compute the signal quality score for the record by comparing each beat or pulse with the previous one, calculating the Pearson correlation coefficient (*r*) defined as Eq. 1; we call this the signal quality index (SQI) score.

**Fig 4:**
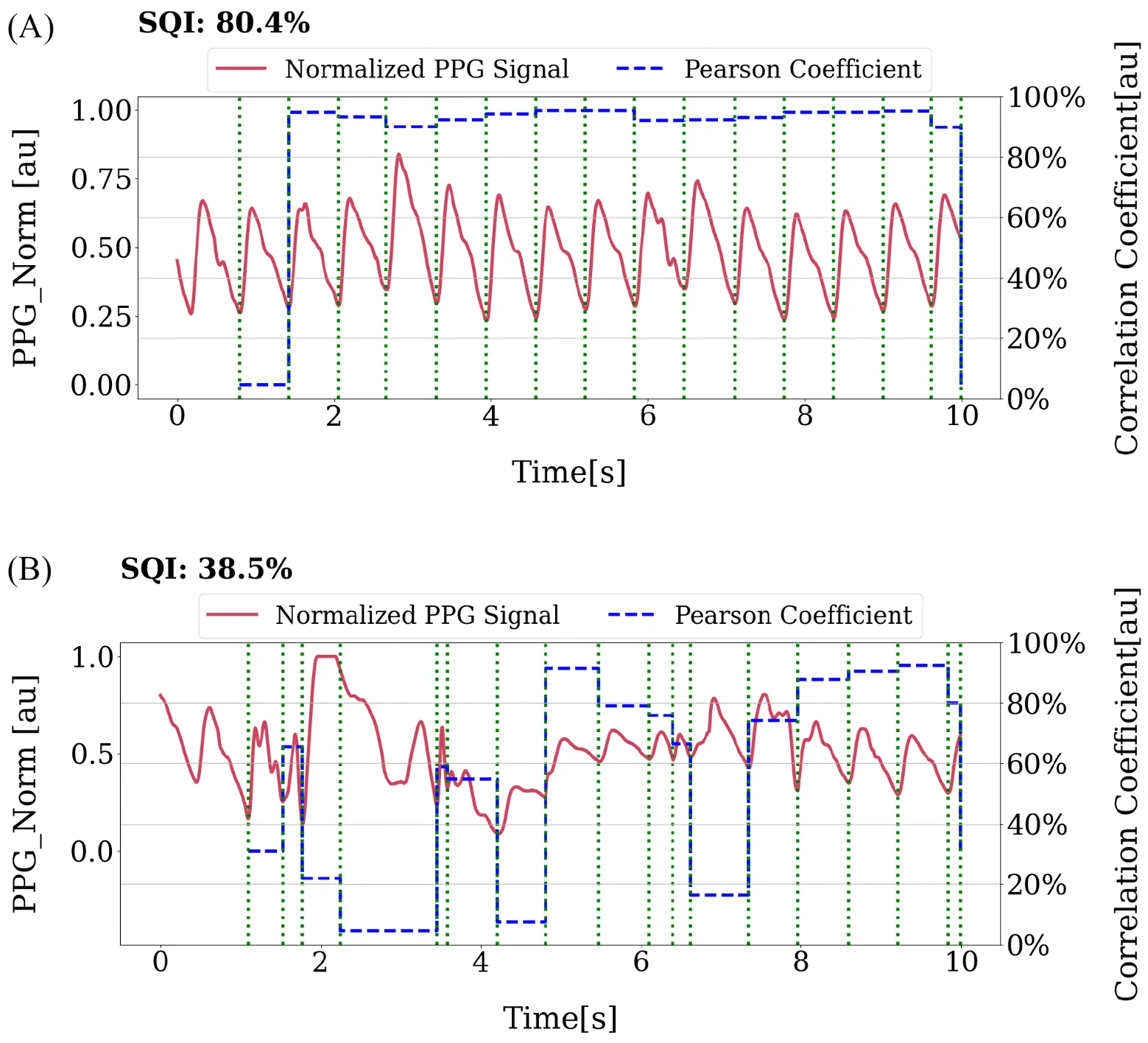
Signal quality assessment. **(A)** Sample subject record with a high SQI score above the set threshold of 70%, indicating that the signal is fit for feature extraction. and **(B)** Same subject, different record (sliding window segment) with a low SQI score. Records with SQI scores below 70% are considered unfit for feature extraction and are excluded from the final curated dataset.


$$\begin{array}{c} r=\frac{\sum_{i=0}^{N-1}[x_{i}-\hat{x}].[y_{i}-\hat{y}]}{\sqrt{\sum_{i=0}^{N-1}[x_{i}-\hat{x}{]}^{2}.\sum_{i=0}^{N-1}[y_{i}-\hat{y}{]}^{2}}} \end{array}$$ (1)

Eq. 1: *r* is the normalized cross-correlation, Pearson’s coefficient [33] between two vectors; $\hat{x}$ and $\hat{y}$ are the empirically determined means of $x_{i}$ and $y_{i}$, respectively; and N is the number of samples in the two time series.

The SQI score, derived from the correlation coefficient *r*, reflects the consistency of the recorded PPG signal. A higher SQI score, as shown in Fig 4(A), indicates strong similarity between successive pulses of a record, and suitability for feature extraction. Conversely, a lower SQI scores, observed in certain records, such as Fig 4(B), suggest the presence of noise or motion artifacts, rendering it unsuitable for feature extraction. To isolate reliable data, we applied an SQI > 70% threshold, following established standards in physiological signal processing [34,35]. Removing or lowering this threshold could force the model to process noisy segments containing high-variance outliers. Thus, a 70% threshold strikes a balance that is stringent enough to exclude major sensor errors and motion artifacts, yet permissive enough to maintain a sufficient dataset for our model.

6. Qualified signals: Each record that successfully passes the signal processing pipeline and satisfies a signal quality index (SQI) threshold greater than 70% is retained for model training and evaluation; this we refer to as a qualified signal. Windows failing to meet this criterion are excluded, as low-quality or noisy signals can distort waveform morphology and degrade BP estimation performance.

### 3.3 Blood pressure estimation

#### 3.3.1 Ground truth definition.

The ground truth/target variables in this analysis were systolic blood pressure (SBP) and diastolic blood pressure (DBP), which are critical indicators of cardiovascular health. These variables were derived from the gold standard arterial blood pressure (ABP) signals, which provide continuous and precise measurements of blood pressure over time. **SBP**, representing the peak pressure in the arteries during the heart’s contraction phase (systole), was determined by identifying the maximum pressure point in the ABP waveform for each heartbeat. **DBP**, reflecting the minimum pressure in the arteries during the heart’s relaxation phase (diastole), was identified as the lowest pressure point in the ABP waveform before the start of the next systolic phase.

#### 3.3.2 Feature extraction.

Features delineating the morphological characteristics of the PPG signals were extracted from fiducial points (points of interest) identified on the pulse wave and its derivatives using established techniques detailed in previous studies [9,36,37]. The feet/onset and systolic peak are simply detected as the minima and maxima, whereas reliable detection of the dicrotic notch and diastolic peak requires the use of derivatives. The first derivative of the Photoplethysmogram (PPG) signal, known as the Velocity Photoplethysmogram (VPG), represents the rate of change of the PPG waveform. It highlights key transitions in the signal, such as the steepness of the rising and falling edges, and provides timing-related features, including the time to peak systole and the intervals between critical points. The second derivative is commonly characterized by five fiducial points [36], spanning the time from early systole to the dicrotic notch. The extracted features included peak amplitudes, dicrotic notch, pulse area, and pulse width measured at specific points of the cardiac cycle, such as 25%, 50%, and 75% of pulse height [38]. We also computed ratios between some of these features to capture additional relationships within the signal. Heart rate and biometric attributes, such as age, were incorporated into the analysis.

#### 3.3.3 Feature selection.

To identify the optimal set of features for our blood pressure (BP) prediction model, we used the Kolmogorov-Smirnov (KS) statistic. This nonparametric test measures the maximum difference between the empirical cumulative distribution functions of two samples, allowing us to assess each feature’s discriminative power. By comparing the distribution of feature values across different BP ranges, we were able to quantify the ability of each feature to differentiate between these ranges. We considered features with higher KS statistics more informative and prioritized them in the feature selection process. This approach ensured that our model utilized the most relevant and statistically significant features, thereby enhancing its predictive accuracy and robustness. We show feature ranking by KS statistics in the Supplementary Material [Fig A in S2 Appendix].

#### 3.3.4 Model selection.

We trained three regression models—Random Forest Regressor (RFR), Support Vector Regression (SVR), and Gradient Boosting Regressor (GBR)—using the curated dataset. We optimized the hyperparameters using Grid Search with 5-fold cross-validation on the training set. The hyperparameter tuning specifications are shown in the Supplementary Material [Table 3 in S2 Appendix]. Model performance was evaluated using Mean Absolute Error (MAE). We selected the configuration with the lowest MAE as the best-performing model. We compared the performance of our selected best-performing model against a benchmark established in [22].

#### 3.3.5 Evaluation metrics.

1. Mean Absolute Error (MAE):

This measure quantifies the average absolute difference between the actual and predicted values


$$\begin{array}{c} MAE=\frac{1}{n}\sum_{i=1}^{n}|y_{i}-{\hat{y}}_{i}| \end{array}$$ (2)

where *n* is the number of data points, $y_{i}$ represents the actual value of the *i*-th data point, ${\hat{y}}_{i}$ represents the predicted value of the *i*-th data point.

2. Root Mean Square Error (RMSE):

is a widely used metric to evaluate the predictive accuracy of a model. It measures the average magnitude of the error between the predicted and actual values, emphasizing larger errors due to its squared error term. RMSE is expressed in the same units as the target variable, making it intuitive for interpretation.


$$\begin{array}{c} \text{RMSE}=\sqrt{\frac{1}{n}\sum_{i=1}^{n}(y_{i}-{\hat{y}}_{i}{)}^{2}} \end{array}$$ (3)

#### 3.3.6 Experimental set-up.

**Training and testing:** We divide the curated dataset into training (80%) and testing (20%) sets. The training set was used for model training, while the testing set served as a held-out dataset for final evaluation, ensuring an unbiased assessment of the model’s performance.

**Demographic tiling:** We segmented the data into distinct demographic tiles, defined by combinations of selected demographic attributes: age group, race, and gender. For example, the subgroup 18–25-Asian-Male represents individuals within the 18–25 age group who identify as Asian males. This approach generated $N_{tile}=n_{\text{age\_group}}\times n_{\text{race}}\times n_{\text{gender}}$ unique subpopulations, where $N_{tile}$ denote the number of categories within each attribute. In our case, this process resulted in $N_{tile}=56$ unique tiles. Each tile functioned as an independent evaluation set, enabling a granular assessment of the model’s consistency and robustness across intersecting demographic groups.

**Cross-validation:** We divide the training set into *K* = 5 equal folds. At each iteration, we trained the model using four folds; the remaining fold served as the validation set. To avoid leakage of a unique subject’s information across folds, we constrained all records belonging to a subject to the same fold. Additionally, we stratify the dataset by intersectional subgroups to maintain their distribution across folds.

### 3.4 Subgroup analysis

**Individual subgroup analysis:** We evaluated the performance of the model within each demographic attribute: age group, race, and sex separately. For each subgroup, we computed performance metrics, including MAE, *R*^2^, and RMSE, to assess variations in precision and error rates. Population groups analyzed include four racial groups: Asian, Black, Hispanic, and White. Two gender categories - female and male. Five age groups - 18–25, 26–35, 36–45, 46–54, and 65–89, as described in Table 1 and Fig 3.

**Intersectional subgroup analysis:** We performed a more granular evaluation of model performance across intersectional subgroups in two and three tiers. The two-way intersection performance evaluation involves the combinations of age group and race (e.g., 18–25-Asian); age group and gender (e.g., 18–25-Female); and race and gender (e.g., Asian-Female). The three-way intersection subgroup analysis evaluates all unique combinations of age group, race, and gender. Each combination, referred to as a demographic tile, represented a specific subpopulation (e.g., 18–25-Asian-Male).

## 4. Results & discussion

We compare our results with previous literature, discuss the impact of signal quality assessment on model accuracy, and analyze model predictions across each demographic subgroup, discussing any performance disparity observed.

### 4.1 Performance comparison to prior works

Several studies have explored different approaches to estimating blood pressure from PPG signals. Common and relevant approaches include signal-to-target [22], which estimates BP values from models trained directly on PPG, and feature-to-target, which extracts features from PPG signals and estimates BP values from those features. In this study, we focus exclusively on the feature-to-target approach, using features extracted from PPG signals with the targets being SBP and DBP values. We compare our findings with selected existing literature that also employed the same database as we did, the MIMIC III waveform database, and used conventional ML models as well as the feature-to-target methodology. Our trained enhanced models (using the WiSQI approach as described in subsection 4.2), in comparison to three existing machine learning-based BP estimation algorithms, demonstrate predictive capabilities that are competitive with state-of-the-art methods. As highlighted in Table 2, our enhanced models achieve an MAE of 3.36 mmHg for SBP and 1.76 mmHg for DBP, both of which are well within the AAMI standard (MAE ≤ 5mmHg).

**Table 2:** This table presents the final evaluation of our trained enhanced models (using the WiSQI approach) against existing conventional machine learning benchmarks for blood pressure prediction. Results are reported for the independent held-out test set to assess the model’s ability to generalize to unseen data.

Our results vs. existing literatures’						
Paper	SBP			DBP		
	R^2^	MAE	RMSE	R^2^	MAE	RMSE
[22]	–	15.86	–	–	7.66	–
[37]	0.88	4.75	–	–	–	–
[24]	0.78	4.17	–	0.72	8.22	–
**Ours: RF**	0.94	3.65	5.05	0.94	1.93	2.65
**Ours: SVR**	**0.95**	**3.36**	**4.98**	**0.94**	**1.76**	**2.58**
**Ours: GBR**	0.93	3.63	5.23	0.93	1.83	2.65

### 4.2 Signal quality assessment

To assess the impact of signal quality on the blood pressure estimation model, we implemented two variations in the preprocessing pipeline. (1) Without-SQI (WoSQI) approach and (2) With-SQI (WiSQI) approach:

- **WoSQI Approach:** In this approach, all records were included for feature extraction and model training, regardless of their signal quality index (SQI) scores.
- **WiSQI Approach:** For this approach, we retained records meeting a 70% SQI threshold for feature extraction and model training.

The results from comparing these approaches are shown in Table 3, which demonstrates a significant performance improvement when comparing the Baseline Models (WoSQI) to the Final Models (WiSQI). This improvement is evident in the reduced values of both MAE and RMSE across all evaluated algorithms, including SVR, RFR, and Gradient Boosting Regression (GBR). For SBP, the Baseline Models displayed MAE values of *9.10 mmHg* for SVR, ***7.27 mmHg*** for RF, and *8.29 mmHg* for GBR. In contrast, the Final Models achieved significantly lower MAE values of *3.51 mmHg* (SVR), ***3.35 mmHg*** (RF), and *3.46 mmHg* (GBR). Similarly, for DBP, the Baseline Models reported MAE values of *4.72 mmHg* (SVR), *7.40 mmHg* (RF), and ***4.28 mmHg*** (GBR). However, the SQI-Integrated Model for DBP produced much lower MAE values of *1.84 mmHg* (SVR), *3.14 mmHg* (RF), and ***1.80 mmHg*** (GBR).

These results show the importance of including signal quality assessment in the preprocessing stage before features are extracted and observing a representative and considerably even demographic distribution during cross-validation splits. We analyzed model performance at the subgroup level in subsubsection 4.3.1.

**Table 3:** Comparative analysis of training methodologies using k-fold cross-validation: Baseline Model (WoSQI, Demographic-Independent) vs. Enhanced Model (WiSQI, Demographic-Aware). The baseline utilizes all available records, whereas the WiSQI approach filters records at a 70% Signal Quality Index (SQI) threshold and enforces demographic-aware partitioning to ensure equitable distribution across folds.

Results of Model Training Approaches: Baseline and Enhanced Model						
	SVR		RF		GBR	
	MAE±SD (mmHg)	RMSE	MAE±SD (mmHg)	RMSE	MAE±SD (mmHg)	RMSE
Baseline Model (SBP)	9.10±0.14	12.56	**7.27±4.18**	**10.38**	8.29±0.16	11.18
Enhanced Model (SBP)	3.51±0.15	5.20	**3.35±3.38**	**4.76**	3.46±0.09	5.02
Baseline Model (DBP)	4.72±0.14	6.83	7.40±2.81	10.34	**4.28±0.12**	**5.92**
Enhanced Model (DBP)	1.84±0.10	2.74	3.14±2.99	4.33	**1.80±0.08**	**2.64**

### 4.3 Enhanced model: Performance analysis by demographic subgroups (SVR)

We evaluated the performance of our best-performing model on three demographic groups: race, age group, and gender. The results are presented below:

**Fig 5:**
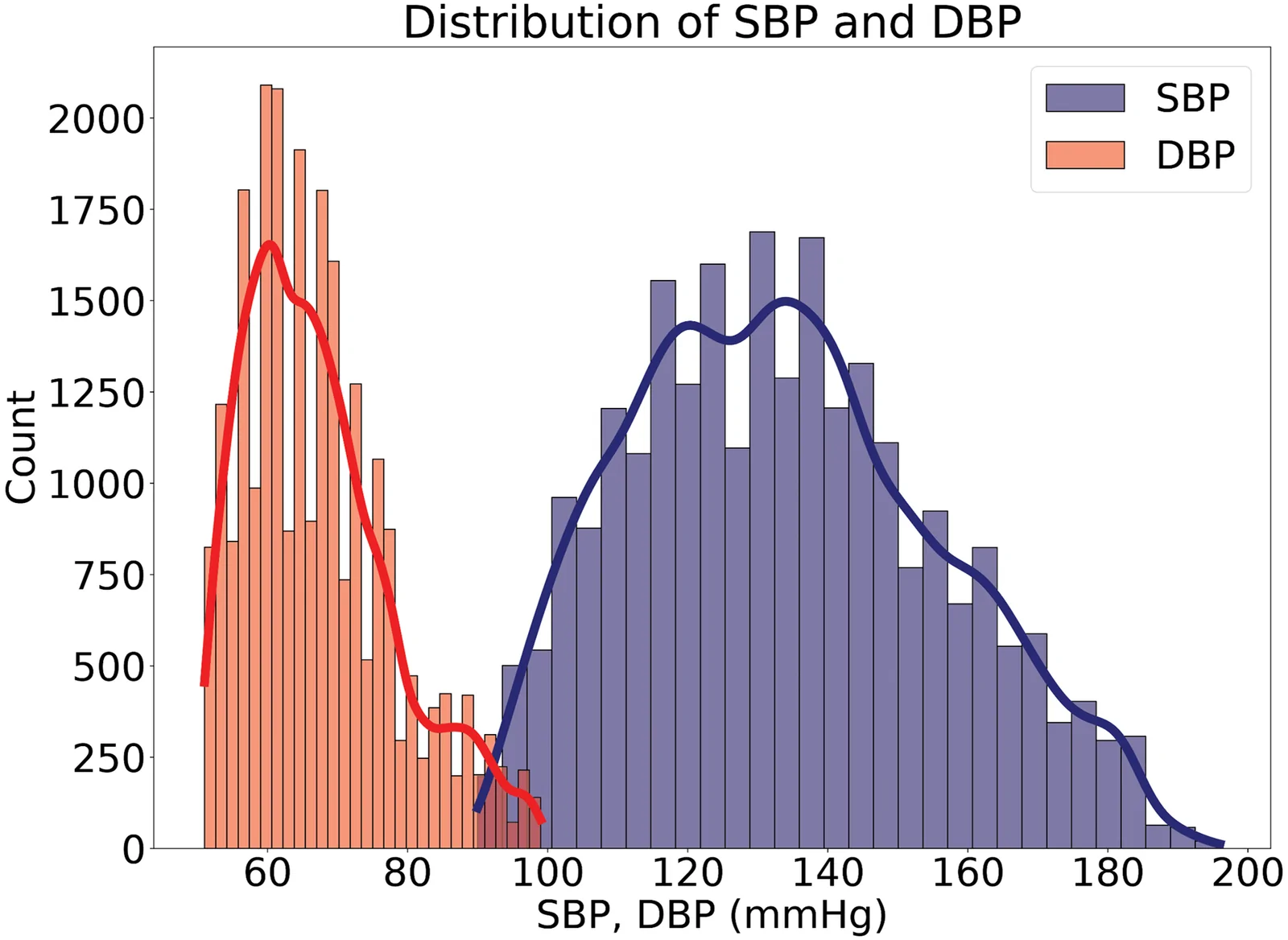
Distribution of the target variables, SBP and DBP from the final curated dataset.

#### 4.3.1 Individual subgroup analysis.

Fig 6 (SBP) and Fig 7 (DBP) illustrate the MAE for each demographic subgroup, while Table 3 directly compares the impact of SVR, RF, and GBR models on SBP within the same subgroup. In general, age seems to be an important factor influencing prediction accuracy. The MAE of SBP and DBP for younger groups (such as 18-25 years old and 26-35 years old) is generally lower, while the error for older groups (especially 56-65 years old and 65-89 years old) is significantly higher. Racial differences are also quite obvious. In many cases, the MAE of Asian and Hispanic subgroups is relatively low, while the error rate of Black and “Other” subgroups is relatively high. Gender differences are slightly less pronounced, but still significant: the MAE of female participants is often slightly lower than that of male participants, indicating that potential physiological or wearable device usage differences can affect PPG waveforms.

**Fig 6:**
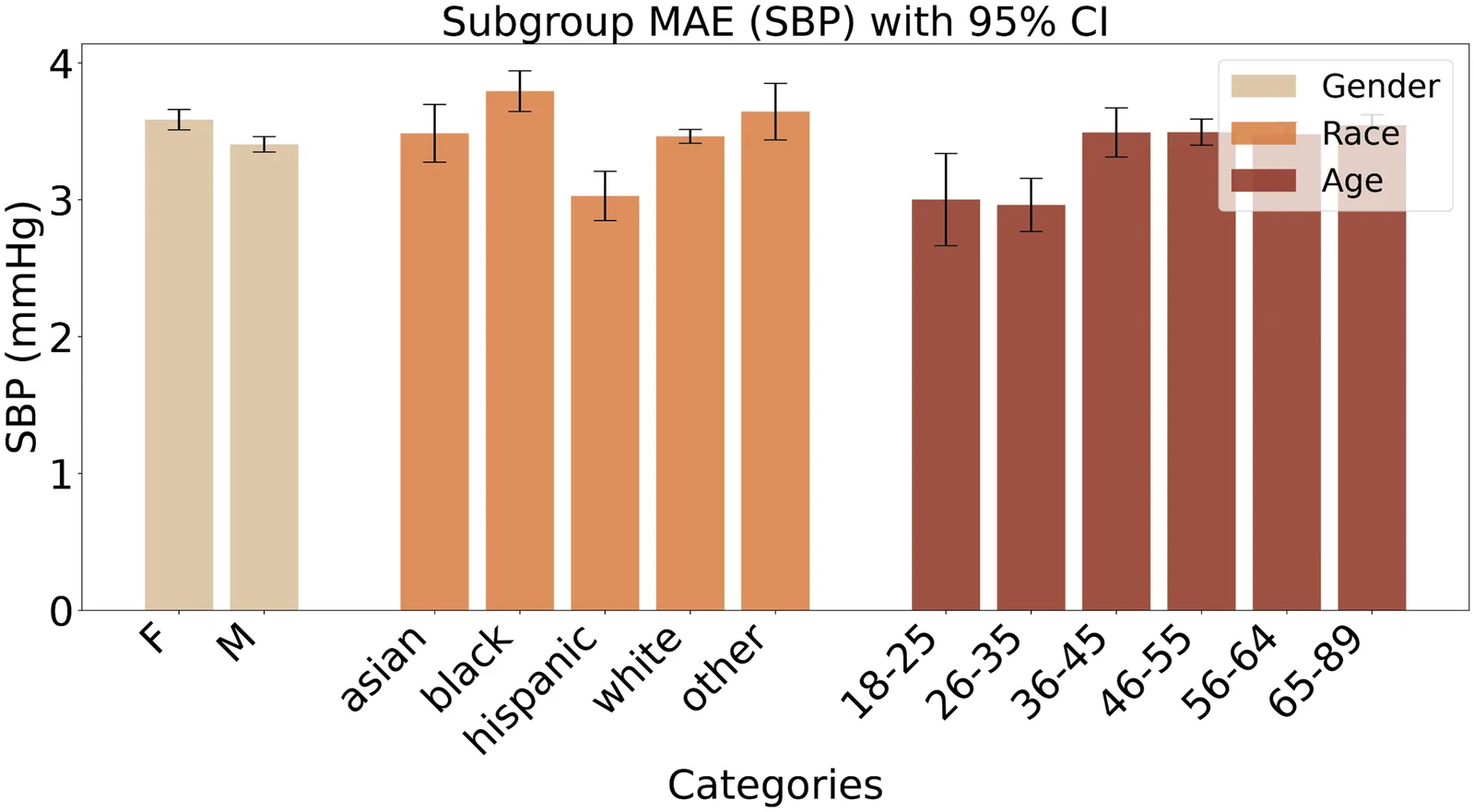
SBP: SVR model performance analysis across demographic subgroups. MAE is lower for younger age groups (ages 18–25 and 26–35) than for older groups (ages 56–65 and 65–89), which show higher error rates. Additionally, the figure shows noticeable racial differences, as the MAE for Asian and Hispanic subgroups is relatively low, whereas Black and “Other” subgroups tend to have higher error rates.

**Fig 7:**
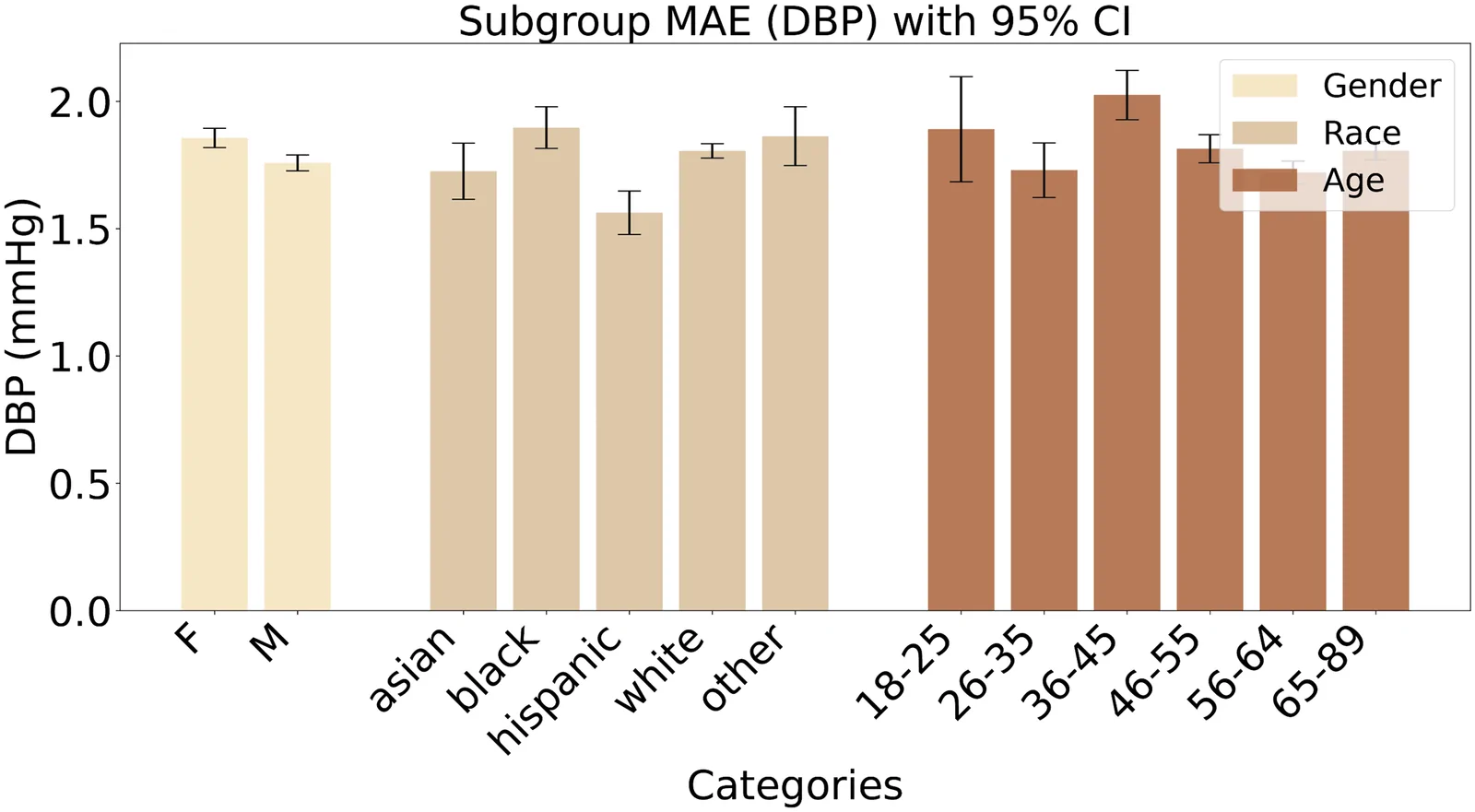
DBP: SVR model performance analysis across demographic subgroups. Model performance for DBP shows a similar MAE trend to SBP above in Fig 6 for the racial categories. However, age groups 18–25 and 36–45 have higher error rates, as depicted by the MAE values.

We evaluated the performance of each model (RF, SVR, GBR) in different demographic subgroups (age group, race, and gender) under WiSQI and WoSQI conditions. Tables 4 and 5 compare the results of SBP and DBP with SQI filtering, while Tables 6 and 7 show the corresponding analysis without filtering.

**Table 4:** SBP: Comparative analysis of the predictive performance of RF, SVR, and GBR models across demographic subgroups.

	SVR		RF		GBR	
	MAE±SD (mmHg)	RMSE	MAE±SD (mmHg)	RMSE	MAE±SD (mmHg)	RMSE
**Age**						
18-25	2.97±2.96	4.19	3.63±2.84	4.61	3.08±2.87	4.21
26-35	**2.81±2.72**	3.91	4.35±3.86	5.82	**2.91±2.84**	4.06
36-45	3.63±4.16	5.52	**3.18±3.13**	4.46	3.53±3.84	5.21
46-55	3.47±3.62	5.02	4.02±4.41	5.97	3.43±3.54	4.93
56-64	3.54±3.69	5.11	3.90±4.03	5.60	3.45±3.54	4.94
65-89	3.57±4.05	5.40	3.61±3.83	5.26	3.52±3.78	5.16
**Gender**						
Female	3.62±4.19	5.54	3.94±4.13	5.71	3.55±3.93	5.30
Male	**3.42±3.53**	4.91	**3.59±3.78**	5.22	**3.38±3.38**	4.78
**Race**						
Asian	3.31±3.65	4.93	4.97±5.41	7.34	3.35±3.67	4.97
Black	3.80±3.90	5.44	4.07±4.28	5.91	3.78±3.74	5.32
Hispanic	**2.82±2.69**	3.90	**3.31±2.77**	4.31	**3.00±2.77**	4.08
White	3.52±3.89	5.25	3.60±3.79	5.23	3.44±3.66	5.02
Other	3.65±3.93	5.37	4.77±4.58	6.62	3.67±3.79	5.28

**Table 5:** DBP: Comparative analysis of the predictive performance of RF, SVR, and GBR models across demographic subgroups.

	SVR		RF		GBR	
	MAE±SD (mmHg)	RMSE	MAE±SD (mmHg)	RMSE	MAE±SD (mmHg)	RMSE
**Age**						
18-25	1.94±1.92	2.74	1.67±1.37	2.16	1.96±1.86	2.70
26-35	**1.69±1.73**	**2.42**	1.98±2.03	2.83	**1.66±1.65**	**2.34**
36-45	2.04±2.21	3.01	2.06±2.19	3.00	2.02±2.16	2.96
46-55	1.86±1.99	2.72	1.87±2.04	2.76	1.82±1.94	2.66
56-64	1.76±1.85	2.55	1.78±1.70	2.46	1.74±1.78	2.49
65-89	1.86±2.12	2.82	**1.67±1.64**	**2.34**	1.80±1.97	2.67
**Gender**						
Female	1.90±2.11	2.84	1.85±1.91	2.66	1.86±2.03	2.75
Male	**1.80±1.95**	**2.66**	**1.72±1.73**	**2.44**	**1.76±1.84**	**2.54**
**Race**						
Asian	1.75±2.01	2.66	2.56±2.65	3.68	1.72±1.97	2.62
Black	1.96±2.20	2.95	1.69±1.62	2.34	1.90±2.08	2.82
Hispanic	**1.56±1.58**	**2.22**	**1.19±1.27**	**1.74**	**1.56±1.50**	**2.16**
White	1.85±2.01	2.73	1.74±1.70	2.43	1.80±1.90	2.62
Other	1.93±2.26	2.97	2.39±2.71	3.61	1.94±2.19	2.93

**Table 6:** SBP(Baseline Model): Subgroup analysis of baseline models RF, SVR, GBR, trained using all available records without enforcing demographic-aware split.

	SVR		RF		GBR	
	MAE±SD (mmHg)	RMSE	MAE±SD (mmHg)	RMSE	MAE±SD (mmHg)	RMSE
**Age**						
18-25	6.54±7.02	9.59	9.52±7.62	12.19	6.99±5.92	9.16
26-35	8.53±7.82	11.57	8.80±7.19	11.36	7.58±7.08	10.38
36-45	9.15±8.62	12.58	10.34±8.43	13.34	8.09±7.44	11.00
46-55	8.91±8.44	12.28	10.03±8.75	13.31	8.23±7.39	11.06
56-64	9.12±8.59	12.53	10.16±9.54	13.94	8.15±7.31	10.95
65-89	9.05±8.84	12.65	10.18±9.06	13.62	8.22±7.58	11.19
**Gender**						
Female	9.11±8.74	12.62	11.12±9.58	14.68	8.45±7.64	11.39
Male	8.91±8.56	12.36	9.30±8.38	12.51	7.94±7.28	10.77
**Race**						
Asian	8.50±8.13	11.76	8.46±7.42	11.25	7.75±7.26	10.62
Black	9.57±9.13	13.22	11.17±9.10	14.41	8.69±8.06	11.85
Hispanic	8.41±7.58	11.32	9.79±8.54	12.99	7.75±6.74	10.27
White	8.89±8.54	12.32	9.98±9.11	13.51	8.05±7.35	10.90
Other	10.42±9.99	14.43	10.66±7.49	13.02	9.21±8.04	12.23

**Table 7:** DBP(Baseline Model): Comparison between the predictive performance of RF, SVR, GBR models across demographic subgroups.

	SVR		RF		GBR	
	MAE±SD (mmHg)	RMSE	MAE±SD (mmHg)	RMSE	MAE±SD (mmHg)	RMSE
**Age**						
18-25	4.35±4.59	6.32	5.13±3.59	6.26	4.19±3.40	5.40
26-35	4.61±4.42	6.39	4.41±3.64	5.72	4.24±3.94	5.78
36-45	4.82±4.48	6.58	5.53±4.93	7.40	4.45±3.86	5.89
46-55	4.88±4.84	6.87	4.76±4.09	6.27	4.46±4.16	6.10
56-64	4.54±4.68	6.52	4.49±4.35	6.25	4.10±3.86	5.63
65-89	4.63±5.06	6.86	4.32±4.18	6.01	4.11±4.11	5.81
**Gender**						
Female	4.90±5.00	7.00	5.25±4.74	7.07	4.43±4.18	6.09
Male	4.50±4.74	6.53	4.07±3.80	5.57	4.04±3.90	5.62
**Race**						
Asian	4.14±4.32	5.99	3.40±3.70	5.03	3.89±3.98	5.57
Black	5.00±5.25	7.25	5.56±4.85	7.38	4.55±4.43	6.35
Hispanic	4.43±4.03	5.99	4.08±3.21	5.19	4.19±3.56	5.49
White	4.62±4.84	6.69	4.55±4.28	6.24	4.16±3.99	5.77
Other	5.30±5.12	7.37	4.09±3.36	5.29	4.51±4.03	6.05

#### 4.3.2 Gender-based differences.

For gender, the patterns in Tables 4 and 5 are less obvious, but still worth noting. Female participants generally had slightly lower MAE than men, suggesting differences in physiology or wearable device use that affect PPG signaling. This gap remained visible regardless of SQI filtering, but was less pronounced than the differences observed by age.

#### 4.3.3 Changes based on age.

From Table 4 (SBP), MAE was consistently lower in younger participants (18–25, 26–35) than in older age groups (56–65, 65–89), suggesting that age-related vascular changes and increased arterial stiffness may complicate PPG-based blood pressure estimates in the older population [39,40]. The DBP in Table 5 shows a similar pattern, where MAE and RMSE generally increase with age. When SQI filtering was not removed, these trends persisted - even with some amplification (Tables 6 and 7). Table 8 further illuminates the age-related generalization challenge. Models trained on older groups tend to produce higher errors when tested on younger groups (and vice versa), underscoring the need for age-diverse training sets.

**Table 8:** Model performance is reported as MAE±SD and RMSE for models trained on specific age groups (e.g., 56-65 and 66-89) and evaluated on distinct age subgroups (18–25, 26–35, etc.). The table captures variations in model accuracy, providing insights into potential biases in generalization across age-based partitions.

	SBP (mmHg)		DBP (mmHg)	
	MAE ± SD	RMSE	MAE ± SD	RMSE
18-25	2.89 ± 2.97	2.89	2.89 ± 2.97	2.89
26-35	2.64 ± 2.64	2.64	2.64 ± 2.64	2.64
36-45	3.03 ± 3.44	3.03	3.03 ± 3.44	3.03
46-55	2.94 ± 3.13	2.94	2.94 ± 3.13	2.94
56-65	1.64 ± 1.82	1.64	1.64 ± 1.82	1.64
66-89	1.63 ± 1.79	1.63	1.63 ± 1.79	1.63

#### 4.3.4 Impact on equity and robustness.

Overall, the SVR remains our best-performing model across most subgroups, but these analyses show that no one model performs consistently well across all demographic categories. Differences in errors by race and age, and to a lesser extent by gender, highlight potential equity issues. In Tables 6 and 7, we observe that the WoSQI data amplify differences in performance by race, suggesting that low-quality signals may disproportionately affect certain subpopulations. In practice, ensuring robust, fair performance may require (1) gathering a more diverse training set that represents the target population, (2) improving signal acquisition (e.g., advanced sensors, personalized calibration), and (3) deploying subgroup perception modeling strategies that account for demographic differences. Finally, Table 8 highlights how the age mismatch between training and testing creates systematic bias, reinforcing the idea that well-delineated training sets and domain adaptation techniques may be critical for reliable, generalizable blood pressure estimates.

#### 4.3.5 Limitation.

One key limitation of this work is the generalizability of our findings from ICU cohorts to healthy populations. Because the data were sourced from critically ill patients, the physiological waveforms used for model training may be affected by hemodynamic instability, medical interventions, and continuous sedation. Consequently, the resulting models may not accurately reflect the normal physiological baseline of healthy individuals.

## 5. Conclusion

This paper presents an improved performance of three regression models for blood pressure prediction based on PPG waveform analysis. We present the first demonstration of cohort distribution during the cross-validation phase based on intersections of subgroup demographic tiles (gender, age, race). We also perform a signal quality assessment of the PPG waveform to mitigate against motion noise that affects waveform morphology. Our results suggest that signal quality and demographic representation during model training play a crucial role in the accuracy and reliability of blood pressure predictions. In the age-group performance analysis, we found lower MAE for subjects aged 18–35 years and higher MAE for older subjects aged 56–89, suggesting that age-related physiological changes may contribute to greater estimation errors. This could also be due to the overall population of each subgroup, since MAE measures the mean error across all data points in each group or category. Furthermore, we observed a noticeable performance variation by race. However, results for all racial categories are within the acceptable clinical threshold of AAMI for the estimation of blood pressure [41].

## Supporting information

S1 Appendix Supplementary Figures A–F and Tables A–N. (PDF)

S2 Appendix Supplementary Figure A and Tables A–D. (PDF)
