# Supplementary material for: Demographic subgroup-aware analysis of blood pressure predictive models
Source: PLOS Digit Health. 2026 Oct 15;5(10):e0001687. doi: 10.1371/journal.pdig.0001687 (PMC13671661; doi:10.1371/journal.pdig.0001687)
Supplement: Supplementary file 1 [file pdig.0001687.s001.pdf]

Supporting Information

A Comparison between the predictive performance of RF, SVR, and GBR enhanced models

R^2 Score Comparison: SVR, RF, and GBR achieve robust R^2 values across all subpopulations, which means that our PPG-based features capture a significant portion of the variance in SBP/DBP. Still, some age and ethnic subgroups do have slightly lower R^2 scores, highlighting the importance of collecting diverse, representative samples and adapting models or feature sets for older or underrepresented groups. These results complement the MAE/RMSE findings in the text, highlighting that SVR is generally the best-performing or near-best-performing algorithm, but the overall difference between the three algorithms remains relatively small across many demographic categories.

R-squared score (R^2): This indicates the proportion of variance in blood pressure data that is explained by the model. A higher R^2 score signifies better model fit to the data.

$$R^2 = 1 - \frac{\sum_{i=1}^{n}(e_i^2)}{\sum_{i=1}^{n}(y_i - \overline{y})^2} \tag{1}$$

Table A. R^2 Score: Comparison between the predictive performance of RF, SVR, and GBR models across demographic subgroups

	SBP			DBP		
	SVR	RF	GBR	SVR	RF	GBR
**Age group**						
18-25	0.95	0.89	0.96	0.96	0.97	0.96
26-35	0.96	0.89	0.97	0.97	0.95	0.97
36-45	0.93	0.94	0.93	0.92	0.95	0.93
46-55	0.93	0.93	0.93	0.95	0.93	0.95
56-64	0.93	0.93	0.93	0.94	0.92	0.94
65-89	0.92	0.94	0.93	0.95	0.95	0.95
**Gender**						
Female	0.94	0.94	0.94	0.93	0.95	0.94
Male	0.95	0.94	0.95	0.94	0.94	0.94
**Race**						
Asian	0.94	0.86	0.94	0.92	0.91	0.92
Black	0.94	0.95	0.95	0.93	0.96	0.93
Hispanic	0.97	0.95	0.97	0.96	0.96	0.96
White	0.94	0.94	0.95	0.94	0.95	0.93
Other	0.96	0.91	0.96	0.93	0.86	0.93

B Random Forest Regression Results

Bland-Altman analysis supports the conclusion that while the bias in our method is small, the accuracy (distribution) of systolic blood pressure estimates leaves room for improvement, especially at high blood pressure values. Future work could explore more sophisticated feature extraction or calibration strategies to further narrow these consistency boundaries.

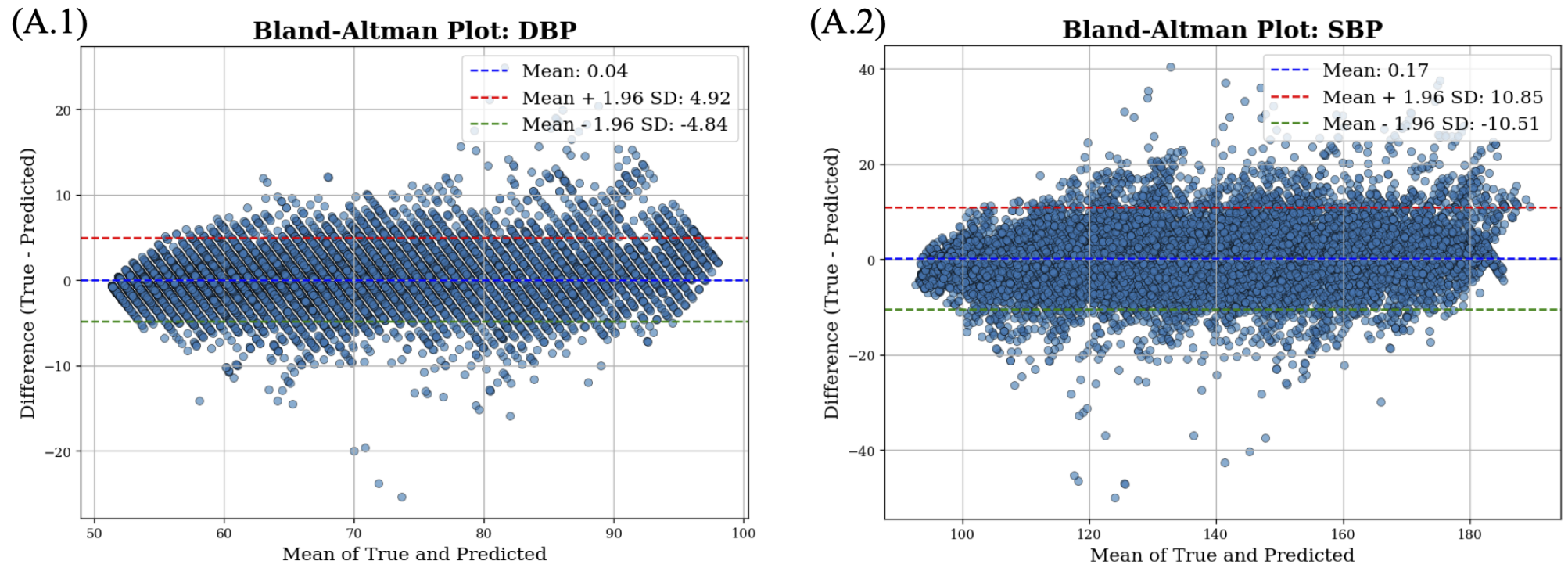

Fig A. Bland-Altman plots for (A.1) SBP and (A.2) DBP. The blue dashed line represents the mean difference, while the red and green dashed lines indicate the limits of agreement (±1.96 standard deviations).

B.1 Individual Subgroup Analysis

The model showed strong predictive power, but systemic differences remained in terms of age - with larger errors in younger groups - and less in terms of race. The gender difference was small, reflecting nearly identical performance. These patterns are consistent with previous findings that certain demographic factors (e.g., age, race) influence PPG-based models. To further improve universality and equity, collecting more diverse and balanced data sets and exploring subgroup-specific calibrations may help close these performance gaps.

Table B. Predictive performance of our blood pressure prediction model across age, racial, and gender groups subgroups

	SBP			DBP		
	R^2 Score	MAE	RMSE	R^2 Score	MAE	RMSE
**Age group**						
18-25	0.80	8.66	9.76	0.46	8.07	8.61
26-35	0.89	5.95	7.20	0.80	5.05	5.76
36-45	0.90	5.38	6.83	0.86	3.58	4.36
46-55	0.94	3.76	5.23	0.81	3.85	4.46
56-64	0.95	3.44	4.71	0.95	1.57	2.17
65-89	0.95	3.89	5.32	0.89	2.78	3.34
**Gender**						
Female	0.96	3.03	4.21	0.97	1.39	1.98
Male	0.97	2.94	4.05	0.97	1.33	1.85
**Race**						
Asian	0.89	5.21	6.75	0.79	3.68	4.32
Black	0.97	3.03	4.09	0.97	1.35	1.85
Hispanic	0.90	6.33	7.37	0.85	3.76	4.22
White	0.97	2.65	3.66	0.98	1.19	1.65
Other	0.92	5.71	7.63	0.93	2.03	2.83

B.2 Intersectional Subgroup Analysis

Tables 10-12 summarize the R^2, MAE, and RMSE of cross-subgroups (i.e., combinations of age × race, age × sex, and race × sex). These results show in more detail how demographic factors work together to influence the accuracy of blood pressure estimates.

Younger, non-White subgroups (e.g., 18–25, Hispanic or 26–35, Asian) occasionally have lower ^{2} and higher errors compared to older cohorts in the same racial group. Within a single age bracket, certain racial subpopulations (e.g., 18–25, White vs. 18–25, Other) may exhibit small but consistent differences in MAE and RMSE—possibly reflecting sample-size imbalances or physiological variations.

Table C. Performance by Age Group and Ethnicity

	SBP			DBP		
	R^2 Score	MAE	RMSE	R^2 Score	MAE	RMSE
**Age group, Race**						
(18-25, asian)	0.98	2.48	3.02	0.96	1.37	1.71
(18-25, hispanic)	0.96	3.58	4.63	0.96	1.58	2.21
(18-25, other)	0.93	4.45	5.42	0.94	1.93	2.31
(18-25, white)	0.91	3.93	6.55	0.95	1.60	2.42
(26-35, asian)	0.93	4.76	6.40	0.94	1.87	2.61
(26-35, black)	0.92	4.31	6.02	0.95	1.94	2.67
(26-35, hispanic)	0.95	3.63	4.93	0.95	1.62	2.24
(26-35, other)	0.97	2.62	3.81	0.97	1.16	1.64
(26-35, white)	0.94	3.79	5.54	0.95	1.71	2.51
(36-45, asian)	0.96	3.55	4.43	0.97	1.47	1.82
(36-45, black)	0.95	3.85	5.25	0.95	1.77	2.53
(36-45, hispanic)	0.93	4.07	5.40	0.95	1.84	2.57
(36-45, other)	0.92	4.40	6.30	0.91	1.99	3.09
(36-45, white)	0.94	3.82	5.53	0.95	1.70	2.44
(46-55, asian)	0.94	3.88	5.25	0.96	1.67	2.30
(46-55, black)	0.94	3.75	5.46	0.94	1.68	2.48
(46-55, hispanic)	0.96	3.48	4.58	0.96	1.53	2.18
(46-55, other)	0.94	3.87	5.36	0.92	1.76	2.67
(46-55, white)	0.94	3.76	5.33	0.95	1.71	2.51
(56-64, asian)	0.95	3.52	5.01	0.94	1.61	2.58
(56-64, black)	0.93	4.14	5.75	0.94	1.81	2.63
(56-64, hispanic)	0.96	3.08	4.01	0.97	1.43	1.86
(56-64, other)	0.93	3.74	5.85	0.94	1.62	2.40
(56-64, white)	0.93	3.98	5.69	0.94	1.78	2.61
(65-89, asian)	0.94	3.87	5.32	0.95	1.68	2.35
(65-89, black)	0.93	3.80	5.81	0.94	1.69	2.54
(65-89, hispanic)	0.93	4.18	5.75	0.94	1.86	2.57
(65-89, other)	0.95	3.46	4.79	0.96	1.59	2.21
(65-89, white)	0.94	3.76	5.36	0.95	1.69	2.45

Young women (e.g., 18-25 years old, female) tend to have slightly lower R^2 values than men in the same age group, which is consistent with the previously observed higher variability among younger participants. In contrast, older women (56-64 years, women or 65-89 years, women) and men (56-64 years, men or 65-89 years, men) performed similarly, with R^2 values typically exceeding 0.90 and moderate MAE values (3-4 mmHg).

At most race-gender intersections, R^2 values remained consistently high (above 0.90 in many cases), but black and Asian subgroups occasionally had slightly lower SBP or DBP accuracy.

Differences in MAE and RMSE between female and male participants in each racial category tended to be small (within 0.3-0.5 mmHg), indicating near parity in model

Table D. Performance by Age-group and Gender

	SBP			DBP		
	R^2 Score	MAE	RMSE	R^2 Score	MAE	RMSE
**Age Group, Gender**						
(18-25, F)	0.83	4.95	8.76	0.92	1.92	2.90
(18-25, M)	0.96	3.45	4.72	0.96	1.52	2.13
(26-35, F)	0.94	3.83	5.57	0.95	1.72	2.51
(26-35, M)	0.94	3.71	5.30	0.96	1.65	2.37
(36-45, F)	0.94	3.78	5.47	0.95	1.71	2.48
(36-45, M)	0.94	3.95	5.57	0.94	1.75	2.50
(46-55, F)	0.94	3.72	5.20	0.95	1.69	2.49
(46-55, M)	0.94	3.78	5.38	0.95	1.69	2.48
(56-64, F)	0.94	3.85	5.54	0.94	1.74	2.57
(56-64, M)	0.93	4.01	5.73	0.94	1.77	2.59
(65-89, F)	0.94	3.82	5.46	0.95	1.70	2.45
(65-89, M)	0.94	3.72	5.33	0.95	1.69	2.45

performance for most race-gender combinations.

Table E. Performance by Ethnicity and Gender

	SBP			DBP		
	R^2 Score	MAE	RMSE	R^2 Score	MAE	RMSE
**Race, Gender**						
(asian, F)	0.95	3.80	5.21	0.96	1.63	2.30
(asian, M)	0.94	3.74	5.18	0.94	1.67	2.45
(black, F)	0.94	3.86	5.53	0.95	1.72	2.52
(black, M)	0.93	3.96	5.84	0.94	1.76	2.60
(hispanic, F)	0.94	3.86	5.25	0.95	1.72	2.40
(hispanic, M)	0.96	3.58	4.78	0.96	1.60	2.24
(other, F)	0.94	3.60	5.28	0.94	1.63	2.47
(other, M)	0.94	3.80	5.48	0.94	1.68	2.40
(white, F)	0.94	3.82	5.49	0.95	1.72	2.51
(white, M)	0.94	3.82	5.46	0.95	1.71	2.49

B.3 Performance Evaluation of Age-Specific Models

We assessed the performance of models trained on specific age groups, revealing significant variations in accuracy. For the youngest subgroup (18-25 years), both SBP and DBP demonstrated a MAE of 2.89mmHg and a RMSE of 2.89. However, as age increased, model accuracy improved. Individuals aged 56-65 exhibited the lowest MAE of 1.64±1.82 and RMSE of 1.64, closely followed by the 66-89 age group with an MAE and RMSE of 1.63±1.79. Conversely, middle-aged groups (36-45 and 46-55) showed worse performance, with MAE values of 3.03±3.44 and 2.94±3.13, respectively. These findings suggest potential biases in model generalization based on age, highlighting the need for age-specific training to enhance accuracy. Overall, optimizing predictive performance for younger and middle-aged populations is crucial, as age significantly influences blood pressure modeling outcomes. Further investigation is warranted to improve results across all demographic segments.

Table M reiterates that no single factor (age, race, or gender) can fully determine the level of error; Instead, the cross-combination determines how consistently the model

Table F. Model performance is reported as MAE±SD and RMSE for models trained on specific age groups (e.g., 56-65 and 66-89) and evaluated on distinct age subgroups (18–25, 26–35, etc.). The table captures variations in model accuracy, providing insights into potential biases in generalization across age-based partitions.

	SBP (mmHg)		DBP (mmHg)	
	**MAE±SD**	**RMSE**	**MAE±SD**	**RMSE**
18-25	2.89±2.97	2.89	2.89±2.97	2.89
26-35	2.64±2.64	2.64	2.64±2.64	2.64
36-45	3.03±3.44	3.03	3.03±3.44	3.03
46-55	2.94±3.13	2.94	2.94±3.13	2.94
56-65	1.64±1.82	1.64	1.64±1.82	1.64
66-89	1.63±1.79	1.63	1.63±1.79	1.63

Table G. Performance by Age Group, Race, and Gender

	SBP		DBP	
**Age Group, Race, Gender**	MAE±SD (mmHg)	RMSE	MAE±SD (mmHg)	RMSE
(18-25, asian, F)	1.39±1.58	1.58	0.80±0.96	0.96
(18-25, asian, M)	2.54±2.35	3.48	0.88±0.95	1.28
(18-25, hispanic, M)	2.39±1.42	2.96	1.07±0.83	1.32
(18-25, other, F)	2.48±2.35	3.02	1.81±0.98	2.16
(18-25, other, M)	2.63±2.79	2.79	1.52±0.73	1.58
(18-25, white, F)	2.07±3.00	3.00	1.45±1.87	1.86
(18-25, white, M)	4.40±3.50	5.62	2.46±1.90	3.11
(26-35, asian, F)	3.20±3.68	3.68	1.89±2.13	2.13
(26-35, black, F)	1.77±2.30	2.30	0.89±1.10	1.10
(26-35, hispanic, F)	6.25±2.15	6.61	2.03±1.47	2.50
(26-35, hispanic, M)	4.05±2.10	5.80	1.92±0.55	2.65
(26-35, other, F)	1.98±2.68	2.68	0.81±1.15	1.15
(26-35, other, M)	1.49±1.14	1.88	0.79±0.61	1.00
(26-35, white, F)	6.00±3.20	6.80	2.74±1.96	3.37
(26-35, white, M)	3.75±3.72	5.29	2.05±2.27	3.06
(36-45, asian, F)	8.16±3.22	8.77	3.69±1.45	3.96
(36-45, black, F)	3.38±3.38	4.68	1.56±1.93	2.23
(36-45, hispanic, F)	1.93±1.60	2.50	1.21±0.93	1.52
(36-45, other, M)	9.51±6.04	11.27	6.69±3.75	7.67
(36-45, white, F)	3.03±2.74	4.09	1.86±1.64	2.48
(46-55, asian, F)	10.97±6.71	12.86	4.69±2.98	5.56
(46-55, black, F)	1.20±0.98	1.55	0.85±0.50	0.99
(46-55, hispanic, M)	1.22±1.00	1.58	0.77±0.49	0.91
(46-55, other, F)	4.69±0.66	4.74	2.04±0.36	2.07
(56-64, black, F)	5.04±4.36	6.66	2.19±1.76	2.81
(56-64, other, F)	4.77±4.86	6.81	1.60±1.47	2.18
(56-64, white, F)	3.44±3.00	4.57	1.67±1.62	2.33
(65-89, asian, F)	2.57±1.85	3.17	1.00±0.62	1.18
(65-89, black, F)	5.38±6.59	8.51	1.87±1.69	2.52
(65-89, hispanic, F)	1.90±1.13	2.21	0.97±0.98	1.38
(65-89, white, F)	3.62±3.60	5.10	1.52±1.57	2.19

estimates systolic and diastolic blood pressure. Targeted data collection: Subgroups with significantly higher MAE or RMSE (e.g., older black/Hispanic women) may require additional samples or more targeted calibration.

Fairness and robustness: While many crossover units show good performance, the presence of outliers underscores the importance of diverse, balanced data sets and possibly specific model improvements for subgroups to ensure fair accuracy across the population.

C Subgroup Analysis of Baseline Model

In contrast to the enhanced model trained based on the WiSQI approach and demographic distribution-aware cross-validation splits, the baseline model was developed using all available records without enforcing demographic information in the cross-validation splits.

Table H. SBP(Baseline Model): Comparison between the predictive performance of RF, SVR, GBR models across demographic subgroups

	SVR		RF		GBR	
	MAE±SD (mmHg)	RMSE	MAE±SD (mmHg)	RMSE	MAE±SD (mmHg)	RMSE
**Age**						
18-25	6.54±7.02	9.59	9.52±7.62	12.19	6.99±5.92	9.16
26-35	8.53±7.82	11.57	8.80±7.19	11.36	7.58±7.08	10.38
36-45	9.15±8.62	12.58	10.34±8.43	13.34	8.09±7.44	11.00
46-55	8.91±8.44	12.28	10.03±8.75	13.31	8.23±7.39	11.06
56-64	9.12±8.59	12.53	10.16±9.54	13.94	8.15±7.31	10.95
65-89	9.05±8.84	12.65	10.18±9.06	13.62	8.22±7.58	11.19
**Gender**						
Female	9.11±8.74	12.62	11.12±9.58	14.68	8.45±7.64	11.39
Male	8.91±8.56	12.36	9.30±8.38	12.51	7.94±7.28	10.77
**Race**						
Asian	8.50±8.13	11.76	8.46±7.42	11.25	7.75±7.26	10.62
Black	9.57±9.13	13.22	11.17±9.10	14.41	8.69±8.06	11.85
Hispanic	8.41±7.58	11.32	9.79±8.54	12.99	7.75±6.74	10.27
White	8.89±8.54	12.32	9.98±9.11	13.51	8.05±7.35	10.90
Other	10.42±9.99	14.43	10.66±7.49	13.02	9.21±8.04	12.23

Table I. DBP(Baseline Model): Comparison between the predictive performance of RF, SVR, and GBR models across demographic subgroups

	SVR		RF		GBR	
	MAE±SD (mmHg)	RMSE	MAE±SD (mmHg)	RMSE	MAE±SD (mmHg)	RMSE
**Age**						
18-25	4.35±4.59	6.32	5.13±3.59	6.26	4.19±3.40	5.40
26-35	4.61±4.42	6.39	4.41±3.64	5.72	4.24±3.94	5.78
36-45	4.82±4.48	6.58	5.53±4.93	7.40	4.45±3.86	5.89
46-55	4.88±4.84	6.87	4.76±4.09	6.27	4.46±4.16	6.10
56-64	4.54±4.68	6.52	4.49±4.35	6.25	4.10±3.86	5.63
65-89	4.63±5.06	6.86	4.32±4.18	6.01	4.11±4.11	5.81
**Gender**						
Female	4.90±5.00	7.00	5.25±4.74	7.07	4.43±4.18	6.09
Male	4.50±4.74	6.53	4.07±3.80	5.57	4.04±3.90	5.62
**Race**						
Asian	4.14±4.32	5.99	3.40±3.70	5.03	3.89±3.98	5.57
Black	5.00±5.25	7.25	5.56±4.85	7.38	4.55±4.43	6.35
Hispanic	4.43±4.03	5.99	4.08±3.21	5.19	4.19±3.56	5.49
White	4.62±4.84	6.69	4.55±4.28	6.24	4.16±3.99	5.77
Other	5.30±5.12	7.37	4.09±3.36	5.29	4.51±4.03	6.05

D Racial categorization

Asian	Black	Hispanic
Asian	Black/African	Hispanic/Latino
Asian - Asian Indian	Black/African American	Hispanic/Latino - Cuban
Asian - Chinese	Black/Cape Verdean	Hispanic/Latino - Dominican
Asian - Filipino	Black/Haitian	Hispanic/Latino - Guatemalan
Asian - Japanese		Hispanic/Latino - Honduran
Asian - Korean		Hispanic/Latino - Puerto Rican
Asian - Cambodian		Hispanic/Latino - Salvadoran
Asian - Vietnamese		
**White**	**Other**	
White	Middle Eastern	
White - Brazilian	Multi Race Ethnicity	
White - Eastern European	Native Hawaiian Or Other Pacific Islander	
White - Other European	Other	
White - Russian	Portuguese	

Table J. Granular details on racial categorization including a breakdown of our selected cohort by demographic subgroups such as race, age, and gender.

E Subgroup Analysis Discussion

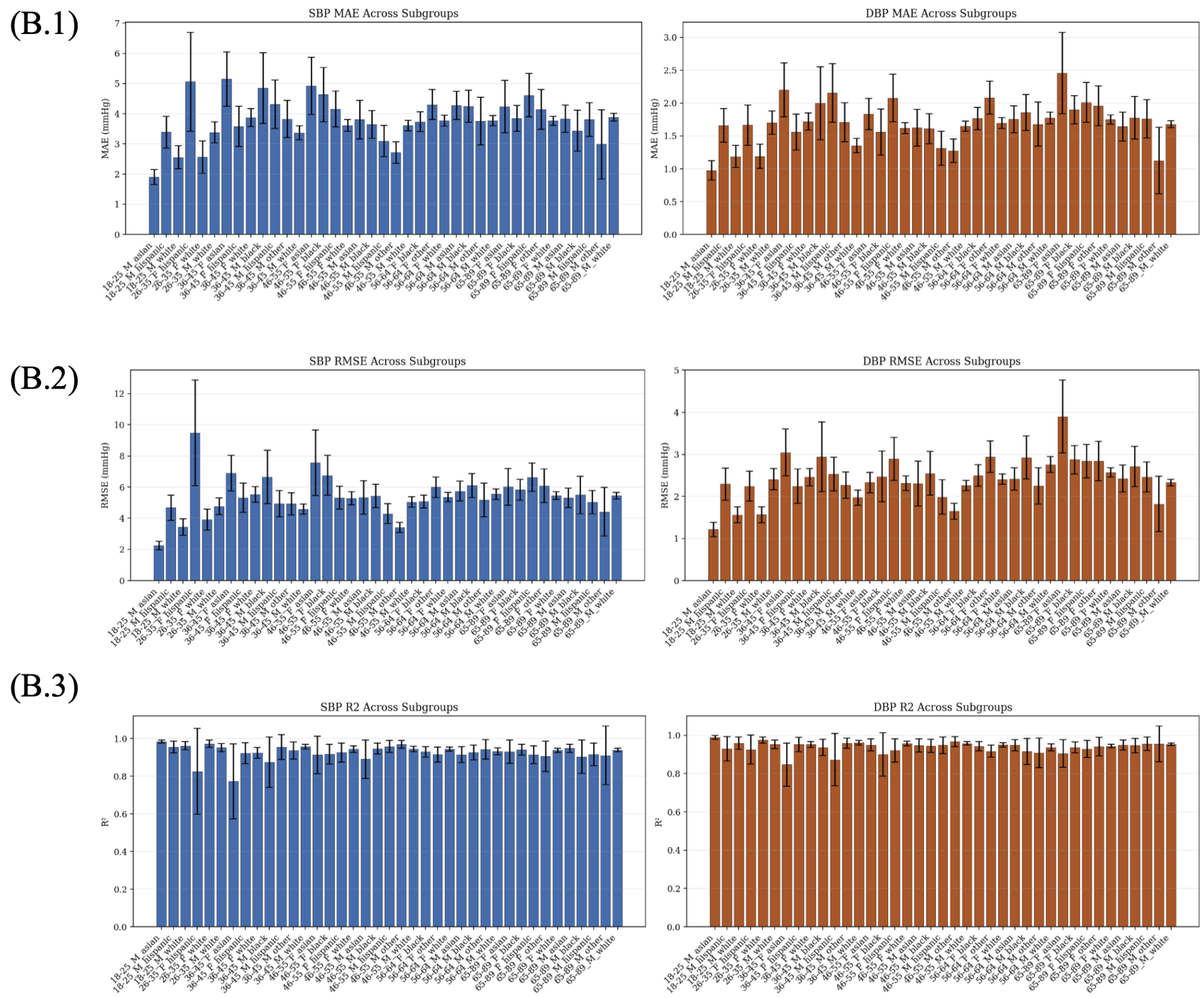

Fig B. This figure presents: (B.1) the mean absolute error (MAE), (B.2) the root mean square error (RMSE), and (B.3) the R^2 of systolic blood pressure (SBP) and diastolic blood pressure (DBP) across different age groups. The results show that prediction errors slightly increase with age, particularly in older groups (56-64 and 65-89), suggesting that model performance may vary depending on age-related physiological changes.

Table K. Overall Model Performance Comparison

Metric	Model A (with age)	Model B (without age)
MAE	2.65	2.65
RMSE	4.06	4.05
R^2 (SBP)	0.948	0.948
R^2 (DBP)	0.9532	0.9532

The subgroup analysis results of this study revealed the performance of the predictive model in different demographic groups and provided some important insights. Overall, the benchmark model (Model A) achieved high accuracy in the SBP prediction task, with MAE = 2.65, RMSE = 4.05, and $R^2 \approx 0.948$. Compared with Model B, which did not include age information, the overall performance of Model A changed little, indicating that the age factor has a relatively limited impact on blood pressure prediction, while other physiological and demographic characteristics may play a more important role in the prediction task.

From the perspective of age groups, the youngest group (18–25 years old) has the

Table L. Subgroup Performance Analysis for Model A (with age)

	MAE (mmHg)		RMSE		R^2	
**Group**	SBP	DBP	SBP	DBP	SBP	DBP
**Age Group**						
18-25	2.37	1.33	3.30	1.83	0.91	0.97
26-35	3.34	1.65	4.59	2.31	0.90	0.94
36-45	2.86	1.61	4.30	2.33	0.92	0.94
46-55	2.65	1.63	4.18	2.31	0.94	0.94
56-64	2.68	1.76	4.00	2.62	0.94	0.94
65-89	2.55	1.74	3.95	2.52	0.95	0.96
**Gender**						
Male (M)	2.53	1.66	3.84	2.41	0.95	0.95
Female (F)	2.80	1.75	4.30	2.54	0.94	0.96
**Ethnicity**						
White	2.52	1.68	3.80	2.43	0.95	0.95
Asian	4.25	1.77	6.20	2.56	0.83	0.91
Other	3.33	1.82	5.10	2.59	0.91	0.89
Hispanic	1.89	1.75	2.62	2.47	0.97	0.97
Black	2.89	1.78	4.65	2.70	0.95	0.96

Table M. Subgroup Performance Analysis for Model B (without age)

	MAE (mmHg)		RMSE		R^2	
**Group**	SBP	DBP	SBP	DBP	SBP	DBP
**Age Group**						
18-25	2.41	1.33	3.35	1.83	0.91	0.97
26-35	3.33	1.65	4.57	2.31	0.90	0.94
36-45	2.85	1.61	4.26	2.33	0.92	0.94
46-55	2.65	1.63	4.18	2.31	0.94	0.94
56-64	2.70	1.76	4.02	2.62	0.94	0.94
65-89	2.55	1.74	3.94	2.52	0.95	0.96
**Gender**						
Male (M)	2.53	1.66	3.83	2.41	0.95	0.95
Female (F)	2.81	1.75	4.30	2.54	0.94	0.96
**Ethnicity**						
White	2.53	1.68	3.80	2.43	0.94	0.95
Asian	4.22	1.77	6.14	2.56	0.84	0.91
Other	3.31	1.82	5.08	2.59	0.91	0.89
Hispanic	1.89	1.75	2.61	2.47	0.97	0.97
Black	2.92	1.78	4.65	2.70	0.95	0.96

Table N. SBP Errors - Younger vs. Older (Model A)

	Value
**Younger Group Size**	393
**Older Group Size**	4767
**Mean (SD) Younger**	4.41 (3.87)
**Mean (SD) Older**	3.60 (3.72)
**T-test Statistic**	3.981
**P-value**	0.0001
**Mann–Whitney U**	1078774.000
**P-value**	0.0000

smallest error, with MAE $\approx$ 2.73, indicating that the model's prediction is most accurate in this age group. This might be because young individuals have relatively stable blood pressure and consistent data distribution. The middle-aged group (26–55 years old) has an MAE ranging from 3.3 to 3.8, showing an increase in prediction error, which may be related to physiological changes, stress, lifestyle, and other factors.

The elderly group (56–89 years old) has the highest error, with MAE $\approx$ 3.8–3.9.

This could be due to the fact that the blood pressure of the elderly is influenced by multiple factors, such as medication use and underlying diseases, which reduces the model's generalization ability in this group.

In terms of gender grouping, the prediction errors for both males and females were relatively close, but the RMSE for females was slightly higher than that for males, indicating that the blood pressure fluctuations in the female group might be greater, affecting the stability of the model. The results of racial grouping showed that the prediction errors for SBP in Asian and Hispanic individuals were relatively large, which might be related to their different blood pressure distribution patterns or relatively smaller sample sizes. Additionally, the R^2 value for the Black group was lower, suggesting that the model's predictive ability for this group was relatively weak, and further optimization of feature engineering or data balancing strategies might be necessary.

From the perspective of the cross-dimensions in subgroup analysis, the triple interaction analysis of age, gender, and race revealed the variations in prediction errors for blood pressure among different populations. For instance, younger Asian males and Hispanic females had higher MAEs, while middle-aged white males had relatively lower MAEs. These findings suggest that in future research and applications, it is advisable to consider optimizing models for specific groups, such as adjusting the weights of training data, incorporating personalized features, or developing group-specific model versions, to enhance the fairness and applicability of predictions.

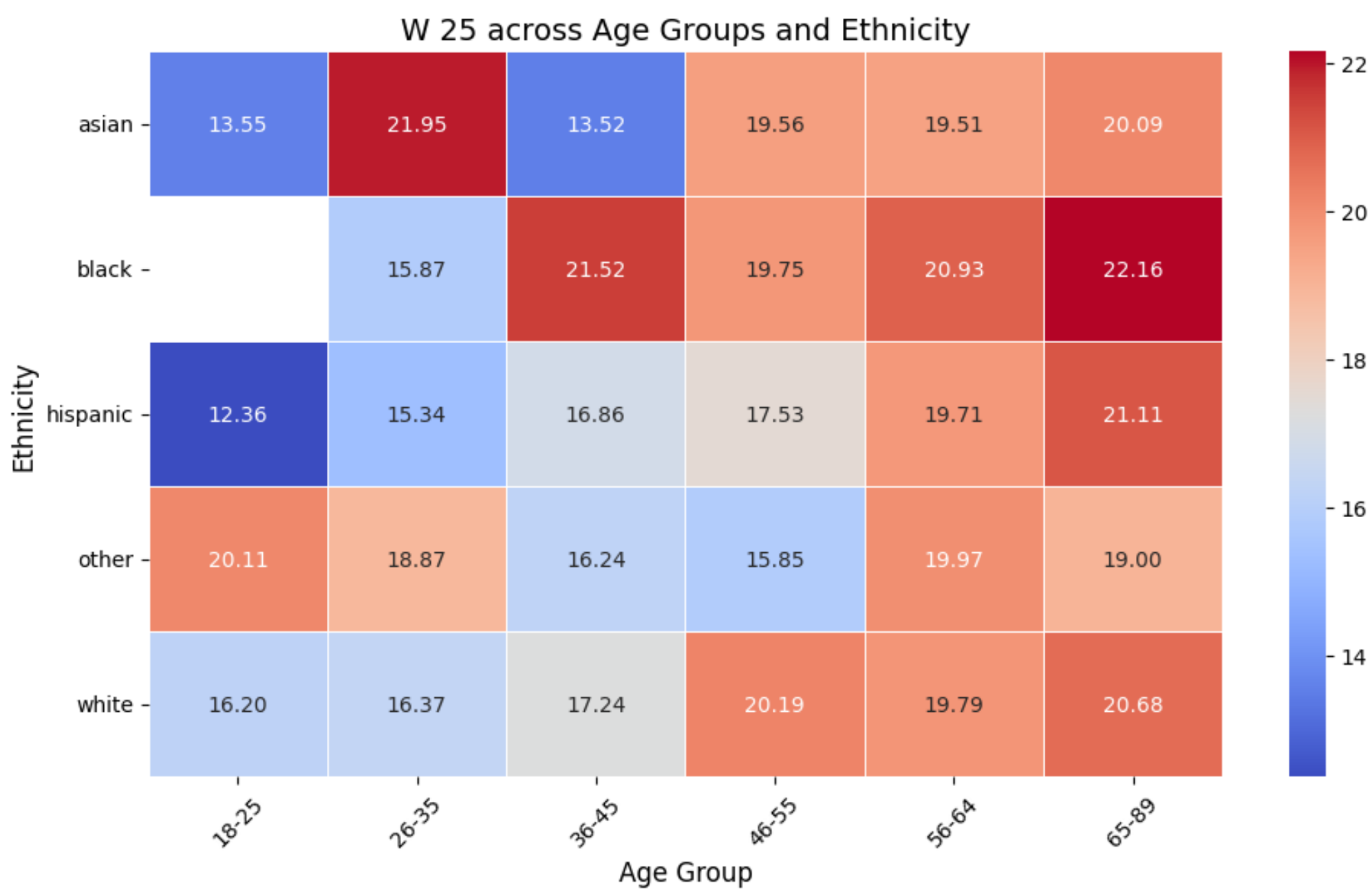

Fig C. Distribution of pulse width at 25% (W25) across different age groups and ethnicities, highlighting variability in waveform characteristics.

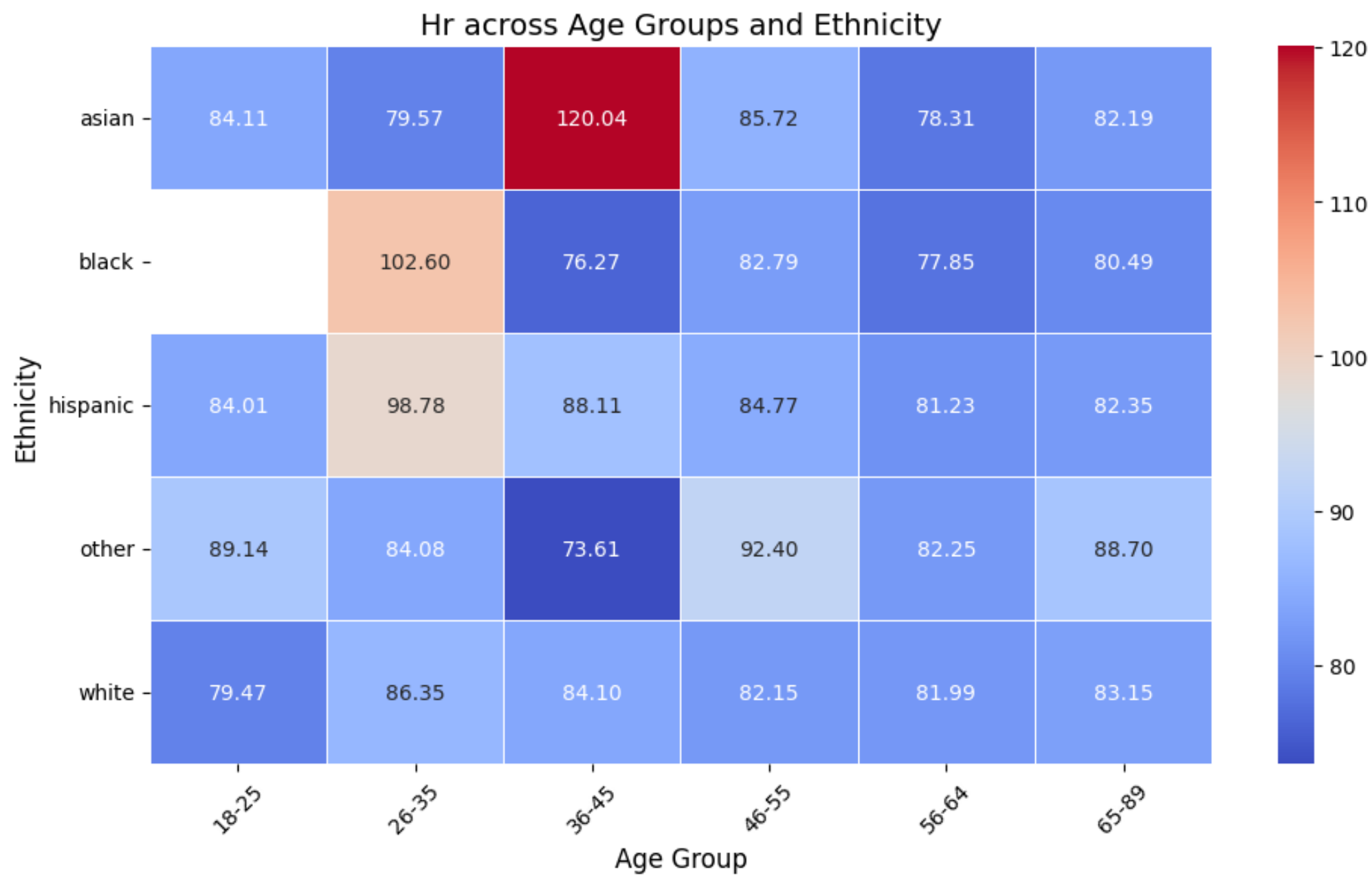

Fig D. Heart rate (HR) distribution across age groups and ethnicities, showing trends in cardiovascular function among different demographic subgroups.

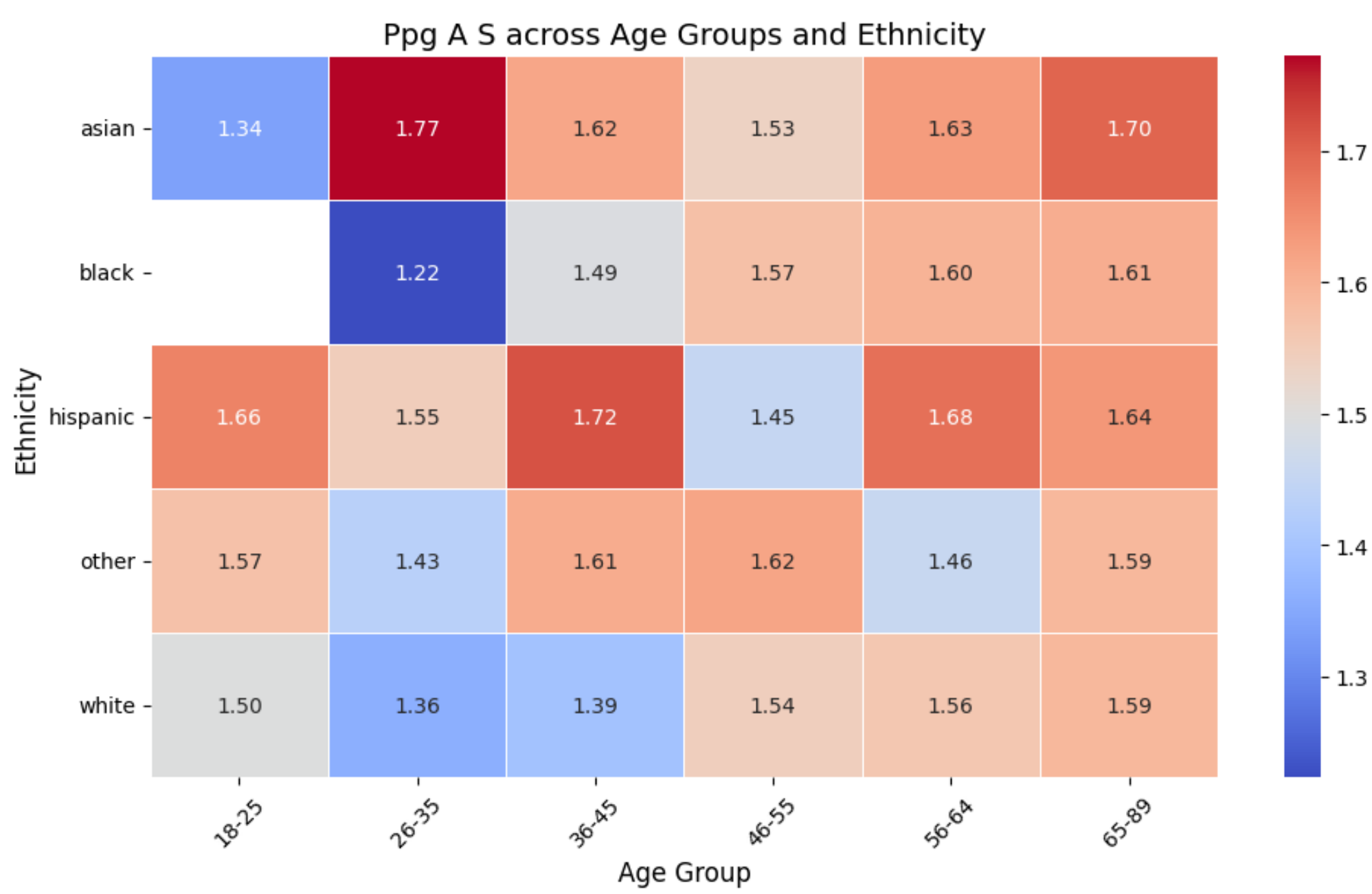

Fig E. Variation of PPG systolic peak amplitude across age and ethnicity, illustrating differences in vascular responses among demographic groups.

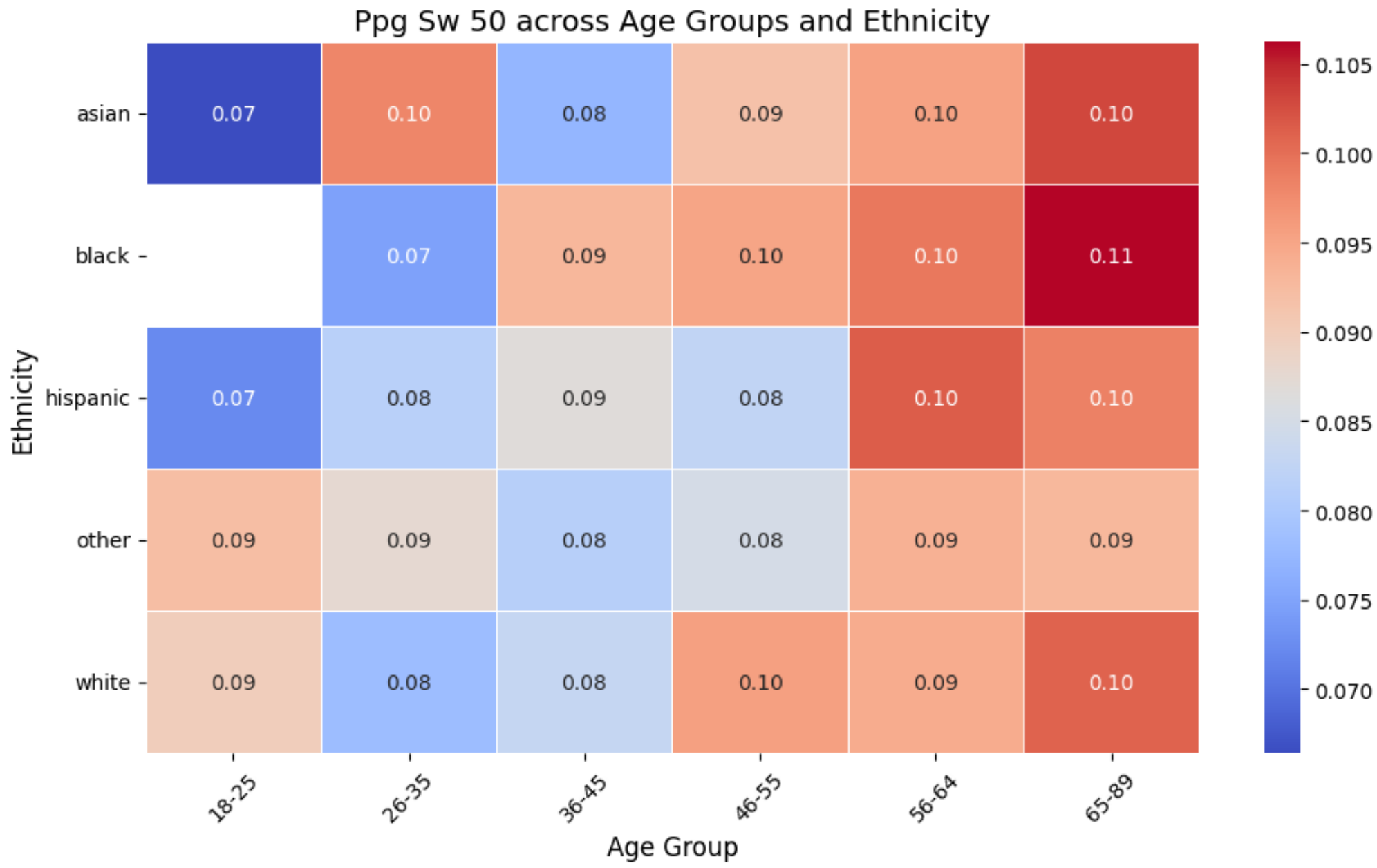

Fig F. Distribution of PPG systolic wave (SW) feature across age groups and ethnicities, capturing the impact of age and ethnicity on PPG waveform characteristics.
