# Supplementary material for: Demographic subgroup-aware analysis of blood pressure predictive models
Source: PLOS Digit Health. 2026 Oct 15;5(10):e0001687. doi: 10.1371/journal.pdig.0001687 (PMC13671661; doi:10.1371/journal.pdig.0001687)
Supplement: Supplementary file 2 [file pdig.0001687.s002.pdf]

Supporting Information

A Description of Features

Table A. Summary of input features used for SBP/DBP prediction. All features are computed from 10 s PPG segments with 50% overlap.

**Feature**	**Definition**	**Unit**
age	Subject chronological age	years
hr	Heart rate estimated from the segment	bpm
w_25	Pulse width measured at 25% amplitude level	seconds (derived)
w_50	Pulse width measured at 50% amplitude level	seconds (derived)
w_75	Pulse width measured at 75% amplitude level	seconds (derived)
pks	Number of detected PPG pulse peaks in the segment	count
ppg_DW_SW_10	Ratio of diastolic width to systolic width at 10% amplitude level	unitless ratio
ppg_DW_SW_25	Ratio of diastolic width to systolic width at 25% amplitude level	unitless ratio
ppg_DW_SW_33	Ratio of diastolic width to systolic width at 33% amplitude level	unitless ratio
ppg_DW_SW_50	Ratio of diastolic width to systolic width at 50% amplitude level	unitless ratio
ppg_DW_SW_66	Ratio of diastolic width to systolic width at 66% amplitude level	unitless ratio
ppg_DW_SW_75	Ratio of diastolic width to systolic width at 75% amplitude level	unitless ratio
ppg_f_2	Frequency-domain PPG feature "f_2" (as defined by the extraction code)	Hz or derived
ppg_median	Median PPG amplitude in the segment	a.u.
ppg_pct_25	25th percentile of PPG amplitude in the segment	a.u.
O_waves_avg	Mean wavelet subband statistic for band "O" (per wavelet features)	a.u. (derived)
S_waves_avg	Mean wavelet subband statistic for band "S"	a.u. (derived)
N_waves_avg	Mean wavelet subband statistic for band "N"	a.u. (derived)
D_waves_avg	Mean wavelet subband statistic for band "D"	a.u. (derived)
w_waves_avg	Mean wavelet subband statistic for band "w"	a.u. (derived)
y_waves_avg	Mean wavelet subband statistic for band "y"	a.u. (derived)
z_waves_avg	Mean wavelet subband statistic for band "z"	a.u. (derived)
a_waves_avg	Mean wavelet subband statistic for band "a"	a.u. (derived)
b_waves_avg	Mean wavelet subband statistic for band "b"	a.u. (derived)
c_waves_avg	Mean wavelet subband statistic for band "c"	a.u. (derived)
d_waves_avg	Mean wavelet subband statistic for band "d"	a.u. (derived)
ppg_a_S	Morphological PPG landmark feature "a" relative to landmark "S" (per extraction definition)	a.u. (derived)
ethnicity	Self-reported ethnicity (one-hot encoded during training; aggregated back to raw feature)	categorical
ppg_t_S	Timing feature to landmark "S" (per extraction definition)	seconds (derived)
ppg_DW_10	Diastolic width at 10% amplitude level	seconds (derived)

B Feature Selection and Ranking

The distribution of heart rate (hr) across different age groups. The heart rate distribution in the younger population (18-25 years old, 26-35 years old) is higher, with an overall median higher than that of other age groups, and there are more outliers, which may be related to higher metabolic rates, exercise frequency, or emotional fluctuations. The heart rate distribution in the middle-aged and elderly population (46-55 years old, 56-64 years old, 65-89 years old) is lower, and the overall trend shows a downward trend. This may be related to changes in cardiovascular function, lifestyle, and the use of antihypertensive drugs.

The distribution of the SBP/DBP ratio (SBP_DBP_ratio) across different age groups. The SBP/DBP ratio increases with age, with the highest ratio in the elderly group (65-89 years old), which may be related to arteriosclerosis in the elderly population, leading to increased systolic blood pressure and decreased diastolic blood pressure. The ratio in younger groups (18-25 years old, 26-35 years old) is relatively lower, indicating better vascular elasticity and more stable blood pressure regulation mechanisms.

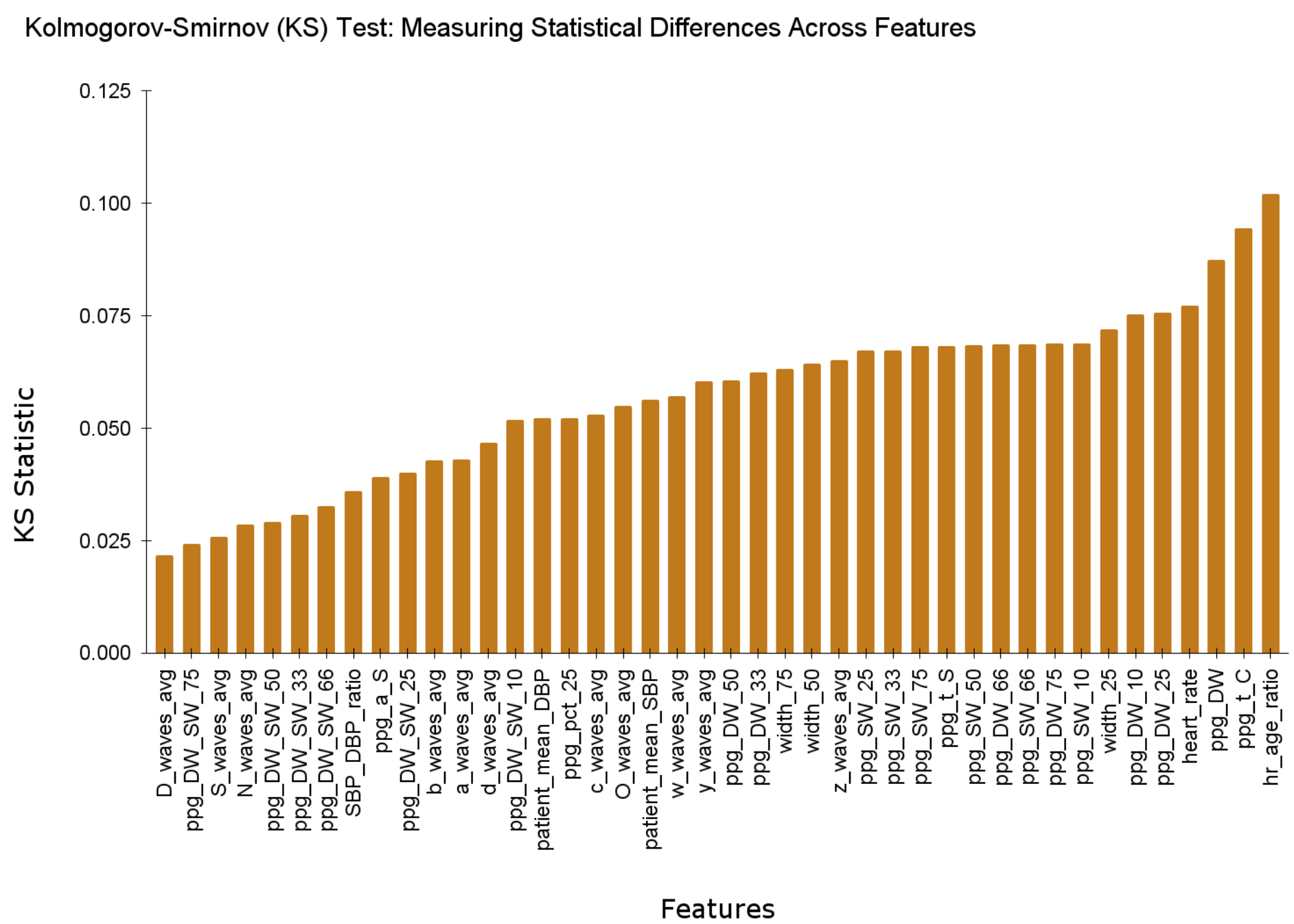

Fig A. This figure represents the KS statistics for various features. The *hr_age_ratio*(0.102) shows the highest distinction, followed by *ppg_t_C*(0.095) and *ppg_DW*(0.087). Several PPG-derived features, such as *ppg_DW*_25, *ppg_DW*_10, and *ppg_SW*_10, also exhibit relatively high KS values. Traditional metrics such as *heart_rate*(0.077) display moderate KS values. Lower-ranked features, such as *D_waves_avg*(0.022) and *S_waves_avg*(0.026), contribute less to differentiation.

The distribution of average systolic blood pressure (patient_mean_SBP) across different age groups. With age, the average systolic blood pressure (SBP) shows an upward trend, especially in the 65-89-year-old group, which is consistent with the age-related trend of hypertension. The SBP distribution in the younger group (18-25 years old) is lower, and the data range is narrower, indicating that the blood pressure of the younger group is relatively stable. The elderly group (65-89 years old) has a larger

dispersion, which may reflect more individual differences in the elderly population, such as the influence of antihypertensive drugs used by hypertensive patients.

The distribution of average diastolic blood pressure (patient_mean_DBP) across different age groups. The DBP distribution in the younger population (18-25 years old) is the highest, but it gradually decreases with age, especially in the 65-89 years old group. The DBP distribution in the elderly group (65-89 years old) is lower and has a larger dispersion, which may be due to arteriosclerosis leading to decreased diastolic blood pressure, or it may be related to the influence of antihypertensive drugs.

The feature selection process strictly adhered to a subject-wise separation to prevent data leakage. The dataset was split into an 80% training/validation set and a 20% completely held-out test set, ensuring that no data from any single subject appeared in both sets. The KS-based feature selection was performed only using the training/validation pool. Specifically, the two-sample KS test evaluated the distribution differences within this training subset to identify the top N features. These features were then used for both the cross-validation training and the final evaluation on the held-out test set. Since the test set was never seen by the KS-selector or the model during training, the final performance metrics (MAE) represent an unbiased evaluation on unseen subjects. In addition, we repeated the analysis with feature selection performed only within each training fold; the result of the analysis is shown in Table B.

Table B. Cross-Validation Performance and Top Features per Fold

**Metric / Fold**	**Fold 1**	**Fold 2**	**Fold 3**	**Fold 4**	**Fold 5**
MAE (mmHg)	3.63	4.07	4.02	3.71	3.94
R^2 Score	0.94	0.93	0.94	0.95	0.94
**Top 10 Features**					
Feature 1	width_25	width_25	width_25	width_25	width_25
Feature 2	c_waves_avg	width_50	age	ppg_t_C	age
Feature 3	ppg_SW_75	age	ppg_t_C	ppg_DW	c_waves_avg
Feature 4	width_50	ppg_DW_25	ppg_DW_75	ppg_SW_75	ppg_SW_75
Feature 5	width_75	width_75	width_50	ppg_SW_66	heart_rate
Feature 6	ppg_SW_66	ppg_DW_66	ppg_SW_75	ppg_DW_75	ppg_DW_75
Feature 7	ppg_DW_25	ppg_DW_75	ppg_DW_66	ppg_SW_50	ppg_DW_25
Feature 8	b_waves_avg	ppg_SW_75	ppg_DW_25	c_waves_avg	ppg_DW
Feature 9	ppg_t_C	ppg_DW_50	ppg_DW	width_50	ppg_DW_66
Feature 10	ppg_DW_75	b_waves_avg	ppg_SW_66	ppg_DW_10	z_waves_avg

Table C. Hyperparameter Tuning Specifications

Model	Hyperparameter	Search Space	Final Optimal Value
SVR	Kernel	[RBF, Poly, Linear]	RBF
	C	[0.001, 0.01, 0.1, 1, 0.25, 10, 100]	1
	epsilon	[0.001, 0.01, 0.1, 0.5, 1.0, 2.0]	0.1
	gamma	[scale, 0.0001, 0.001, 0.01, 0.1, 1, 10]	scale
RBF	n_estimators	[50, 100, 150, 200, 500]	150
	max_depth	[None, 10, 20, 30]	20
	min_samples_split	[2, 3, 5, 10]	3
GBR	learning_rate	[0.01, 0.05, 0.1, 0.2, 0.5]	0.05
	n_estimators	[50, 100, 150]	100
	subsample	[0.5, 0.8, 1.0]	0.8
	max_depth	[3, 5, 10]	5

Table D. Selected Features Summary Statistics (Mean ± SD)

	SBP	DBP	Heart rate	Width25	Width50	Width75	Peak amplitude	Peak time	Peak Cycle
Age Group									
18-25	139.71±22.25	76.18±12.83	81.94±17.99	15.12±4.58	27.8±9.15	51.11±12.88	1.54±0.4	0.18±0.05	0.65±0.24
26-35	126.28±21.95	71.35±12.81	88.9±12.8	16.52±4.54	27.2±6.63	46.61±10.09	1.4±0.37	0.18±0.03	0.56±0.16
36-45	130.24±20.01	70.19±11.49	83.46±17.19	17.6±4.8	28.26±7.45	46.56±11.25	1.44±0.4	0.19±0.04	0.61±0.19
46-55	132.86±21.8	70.6±10.39	83.07±16.19	19.73±5.27	31.52±8.75	50.26±11.59	1.55±0.31	0.2±0.04	0.67±0.17
56-64	132.28±20.48	66.71±9.77	81.31±14.99	19.93±4.49	31.7±7.51	49.94±10.96	1.57±0.33	0.21±0.04	0.68±0.17
65-89	136.34±23.03	64.76±9.96	83.06±15.16	20.73±4.6	32.9±7.34	51.14±9.98	1.6±0.25	0.22±0.04	0.69±0.14
Gender									
Female	133.11±22.25	67.55±10.83	83.81±15.7	20.52±4.7	32.4±7.51	50.28±10.29	1.59±0.27	0.21±0.04	0.68±0.14
Male	134.37±21.84	66.94±10.45	82.08±15.33	19.3±4.95	31.07±8.05	49.98±11.19	1.53±0.33	0.2±0.04	0.66±0.17
Race									
Asian	130.07±20.69	63.55±9.45	82.94±15.39	19.55±4.47	31.06±7.29	49.59±10.53	1.63±0.27	0.21±0.04	0.69±0.16
Black	133.68±22.76	67.24±11.03	80.27±17.56	21.05±5.04	33.98±8.08	53.22±10.29	1.58±0.26	0.21±0.04	0.71±0.17
Hispanic	140.1±23.07	71.28±10.99	85.76±15.93	18.14±5.05	28.79±7.34	46.62±10.39	1.6±0.29	0.2±0.03	0.65±0.15
White	133.49±21.48	67.15±10.5	82.83±15.23	19.91±4.86	31.71±7.84	50.11±10.83	1.55±0.31	0.21±0.04	0.67±0.16
Other	136.31±27.42	67.66±11.02	85.91±14.17	18.48±4.09	29.65±6.78	47.58±10.08	1.55±0.33	0.2±0.04	0.64±0.15

	N_waves_avg	D_waves_avg	w_waves_avg	y_waves_avg	z_waves_avg	a_waves_avg	b_waves_avg	c_waves_avg	d_waves_avg
Age Group									
18-25	0.4±0.45	-0.09±0.29	28.56±4.52	-15.97±4.06	-7.72±6.74	369.27±66.62	-417.1±133.9	252.43±169.72	-237.04±288.99
26-35	0.07±0.43	-0.3±0.27	27.55±2.9	-16.24±4.46	-8.98±7.01	350.85±59.02	-386.54±88.96	251.64±152.37	-233.92±269.48
36-45	0.08±0.41	-0.37±0.36	26.47±3.05	-15.75±3.93	-7.74±6.54	330.15±56.48	-362.6±97.38	204.88±151.74	-157.18±261.7
46-55	0.16±0.34	-0.4±0.33	25.03±3.49	-14.22±3.88	-6.22±4.58	308.61±55.89	-321.14±91.16	175.89±136.65	-110.05±225.94
56-64	0.18±0.33	-0.41±0.33	24.61±3.17	-13.95±3.61	-6.02±4.35	298.12±53.52	-309.21±81.87	157.96±126.87	-78.19±215.41
65-89	0.19±0.3	-0.47±0.31	23.67±3.41	-13.17±3.21	-5.51±2.74	288.52±58.83	-296.64±86.71	135.96±115.88	-55.34±181.24
Gender									
Female	0.17±0.31	-0.48±0.32	23.92±3.55	-13.5±3.31	-5.67±3.3	294.46±61.52	-301.19±93.39	139.27±120.12	-57.57±192.71
Male	0.18±0.35	-0.38±0.33	25.17±3.38	-14.28±3.92	-6.47±4.96	307.36±56.99	-325.91±88.89	178.11±137.91	-114.61±230.84
Race									
Asian	0.17±0.27	-0.48±0.27	24.64±3.29	-13.88±3.49	-5.81±3.66	299.91±53.58	-316.09±87.65	152.73±129.94	-68.48±212.93
Black	0.29±0.3	-0.33±0.35	24.07±3.68	-12.85±3.6	-6.06±3.7	294.31±60.18	-299.6±86.26	139.13±137.63	-74.2±205.54
Hispanic	0.14±0.35	-0.41±0.34	25.87±3.38	-15.14±3.67	-5.85±4.31	310.12±54.77	-328.04±90.19	200.22±152.12	-165.34±244.63
White	0.16±0.34	-0.43±0.32	24.55±3.5	-13.96±3.67	-6.13±4.39	301.41±60.03	-314.58±92.66	159.94±129.0	-85.77±214.01
Other	0.1±0.4	-0.47±0.32	25.31±3.23	-14.64±3.56	-6.47±4.83	312.11±55.16	-335.56±88.32	185.37±127.99	-114.51±232.42
